# Human Umbilical Cord Blood Mononuclear Cells Ameliorate CCl_4_-Induced Acute Liver Injury in Mice *via* Inhibiting Inflammatory Responses and Upregulating Peripheral Interleukin-22

**DOI:** 10.3389/fphar.2022.924464

**Published:** 2022-07-22

**Authors:** Jinming Zhang, Hengben Zhai, Pei Yu, Dabao Shang, Ruidong Mo, Ziqiang Li, Xiaolin Wang, Jie Lu, Qing Xie, Xiaogang Xiang

**Affiliations:** ^1^ Department of Infectious Diseases, Ruijin Hospital, Shanghai Jiao Tong University School of Medicine, Shanghai, China; ^2^ Translational Lab of Liver Diseases, Department of Infectious Diseases, Ruijin Hospital, Shanghai Jiao Tong University School of Medicine, Shanghai, China; ^3^ Department of Orthopedics, Ruijin Hospital, Shanghai Jiao Tong University School of Medicine, Shanghai, China

**Keywords:** cell therapy, liver injury, liver inflammation, liver regeneration, IL-22

## Abstract

**Background:** Human umbilical cord blood mononuclear cells (hUCBMNCs) show therapeutic effects on many inflammatory diseases. The deterioration of acute liver injury is attributed to excessive inflammatory responses triggered by damage-associated molecular patterns (DAMPs) and pathogen-associated molecular patterns (PAMPs). Whether hUCBMNCs treatment is a promising strategy for acute liver injury/failure needs to be investigated.

**Methods:** Liver injury mice induced by PAMPs, DAMPs, or DAMPs plus PAMPs were developed. DAMPs included CCl_4_ (carbon tetrachloride), APAP (acetaminophen), and ConA (Concanavalin A). PAMPs included *Klebsiella pneumoniae* (*K.P.*) and *Salmonella typhimurium* (*S. Typhimurium*). DAMP plus PAMP-induced liver injury was developed by sequential CCl_4_ and *K.P.* administration. hUCBMNCs were injected intravenously.

**Results:** hUCBMNCs significantly prolonged mice survival time in DAMP plus PAMP-induced liver failure but had no benefit in bacteria-infected mice. hUCBMNCs significantly alleviated hepatic necrosis post CCl_4_/ConA insult. In CCl_4_-induced acute liver injury, peripheral levels of interleukin (IL)-22 were upregulated and liver regeneration was enhanced after treating with hUCBMNCs at 48h. The levels of p62 and LC3B-II, autophagy markers, were also upregulated in the hUCBMNC-treated group.

**Conclusion:** hUCBMNCs as a kind of cell therapeutic strategy could attenuate acute liver injury in mice, which is executed by enhancing autophagy and regeneration in the liver via inhibiting inflammatory responses and upregulating peripheral IL-22.

## Introduction

The incidence of acute liver failure (ALF) ranges from 1.4/10 000 (Escorsell et al., 2007) to 5.5/10 000 (Bower et al., 2007) worldwide. It is impossible for the liver to compensate in fulminant liver failure. The only feasible treatment option is liver transplantation (Chew et al., 2020). Thus, new therapeutic strategies for ALF in the reversible stage need to be explored. Inflammatory response and immune-mediated damage of hepatocytes play fatal roles during the deterioration from acute liver injury (ALI) to ALF (Wu et al., 2010). Necrosis of cells triggers the release of cellular contents with some of the endogenous compounds (alarmins) including heat shock proteins, DNA, RNA, and others, called damage-associated molecular patterns (DAMPs), which are able to activate innate immune cells (Scaffidi et al., 2002; Bianchi, 2007). The milieu of inflammation and necrosis in ALF is presumed to predispose patients to infection due to complement deficiency, and/or impaired function of polymorphonuclear or Kupffer cell (Chung et al., 2012). Bacteria release a set of pathogen-associated molecular patterns (PAMPs) such as lipopolysaccharide (LPS), which would be recognized by the innate and acquired immunity system (Bianchi, 2007). DAMPs/PAMPs activate the immune system and generate excessive inflammatory responses, which subsequently aggravate tissue damage and eventually result in liver failure or multiorgan failure (Mihm, 2018; Arroyo et al., 2020).

Cells as a direct therapeutic strategy open a new era for the remedy of many diseases (Yamanaka, 2020). Human umbilical cord blood mononuclear cells (hUCBMNCs) contain a variety of stem cells and low immunogenic immune cells, such as hematopoietic stem cells, endothelial stem cells, lymphoblasts, small embryonic stem cells, mesenchymal stem cells, lymphocytes and bone marrow cells, and can be isolated from human umbilical cord blood conveniently and easily (Weiss and Troyer, 2006). hUCBMNCs have been reported for the immunomodulatory effects on other inflammation-related diseases, such as renal tubulointerstitial fibrosis (Li et al., 2020), inflammation-induced preterm brain injury (Dalous et al., 2013), and LPS-induced acute kidney injury (Paton et al., 2019). However, whether hUCBMNCs have therapeutic effects on ALI/ALF remains to be elucidated. Thus, the present study aimed to investigate the application value and mechanisms of hUCBMNCs treatment in several DAMPs-/PAMPs-induced acute liver injury mouse models.

## Materials and Methods

### Isolation and Identification of hUCBMNCs

The hUCBMNCs were provided by Shandong Cord Blood Bank (Shandong Qilu stem cell engineering Co., Ltd, Jinan, Shandong, China). Human umbilical cord blood samples were collected from healthy newborns with mothers’ consents as described previously (Rubinstein et al., 1995). The blood samples were negative for antibodies against hepatitis B virus, hepatitis C virus, cytomegalovirus, human immunodeficiency virus, and syphilis. hUCBMNCs were isolated from umbilical cord blood by centrifugation on lymphocyte separation medium (Ficoll, d = 1.077 g/ml; Eurobio) (Dalous et al., 2013) and washed twice in Dulbecco’s modified eagle medium (DMEM). hUCBMNCs of 5 × 10^7^ cells were frozen in a sterile 5 ml cryovial with 10% dimethylsulfoxide. hUCBMNCs identified by China National Institutes for Food and Drug Control (NIFDC) showed major expression of CD38, CD4, CD5, and CD3 and expressed no less than 1% of CD34 (Feng et al., 2020). Details for the identification of hUCBMNCs are included in supplementary materials (Supplementary Table S1 and Supplementary Attachment S1) and representative flow cytometry plots of typical cells subsets in hUCBMNCs are shown in Supplementary Figure S1. hUCBMNCs from the same donor were used in each experiment. This study was approved by the Ethics Committee of Ruijin Hospital, Shanghai Jiao Tong University School of Medicine.

### Mouse Experiments

All animal experiments were performed on adult 8-week-old male C57BL/6J mice purchased from Beijing Vital River Laboratory Animal Technology Co., Ltd. Mice were kept in a cage (up to five mice per cage) and maintained in a light (12–12h light-dark cycle) and temperature (24 ± 1°C) controlled room with water and food freely available unless otherwise stated.

For PAMPs-induced liver injury, *Klebsiella pneumoniae* (*K.P.*) (ATCC, 43816, Manassas, United States) (500 CFU/mouse) (Xiang et al., 2020) or *Salmonella typhimurium* (*S. Typhimurium*) (ATCC, 14028s, Manassas, United States) (8 × 10^3^ CFU/mouse) (Ren et al., 2019) was injected intraperitoneally to simulate bacterial infection in mice at appointed time points. For mouse models of DAMPs-induced liver injury, mice were injected intraperitoneally with CCl_4_ (1 μl/g, dissolved in olive oil, v/v 1:4) and APAP [350 mg/kg, dissolved in phosphate-buffered solution (PBS)] (Bird et al., 2018). For ConA-induced acute liver injury model, mice were injected intravenously with ConA (12 mg/kg, dissolved in PBS) (He et al., 2021). For DAMPs plus PAMPs-induced liver injury model, CCl_4_ (0.4 μl/g, dissolved in olive oil, v/v 1:4) and *K.P.* (500 CFU/mouse) were administrated sequentially (Xiang et al., 2020). IL-22 neutralizing antibody (R&D system, Minnesota, United States) (PBS as carrier) was administrated intravenously 2 h before CCl_4_ injection in hUCBMNCs-treated mice (1μg/mouse/time point) (Ji et al., 2017), as well as 20 and 44 h post CCl_4_ injection. Mice administrated with PBS served as a control group. All mouse experiments were approved by the *Institutional Animal Care and Use Committee of Ruijin Hospital*, *Shanghai Jiao Tong University School of Medicine* (Supplementary Attachment S2)*.*

### Treatments of hUCBMNCs in Mouse Models

hUCBMNCs (8 × 10^6^ cells/ml) suspended in PBS were prepared in advance. In CCl_4_ plus *K.P.*-induced liver injury mouse model, CCl_4_ and *K.P.* were administrated intraperitoneally at 0 and 24 h, and hUCBMNCs (1 × 10^6^ cells/mouse) were administrated intravenously at 22 h for pre-treatment or 26 h for treatment. hUCBMNCs were also used to treat the bacterial infection-induced liver injury with *K.P.* or *S. Typhimurium*. In CCl_4_- or APAP-induced acute liver injury mouse models, mice were injected intravenously with hUCBMNCs (1 × 10^6^ cells/mouse) or PBS (as a control treatment) 2 hours (2 h) post insult. Blood and liver tissues were collected at 24, 48, and 72 h post CCl_4_ insult and mice were injected intraperitoneally with BrdU (Bromodeoxyuridine/5-bromo-2′-deoxyuridine) 2 h before they were sacrificed. In APAP model, blood and liver tissues were collected at 6, 12, 24, and 48 h post APAP injection. In ConA-induced acute liver injury model, mice were injected intravenously with hUCBMNCs (1 × 10^6^ cells/mouse) or PBS (as control) 1 h post ConA injection and were sacrificed for the collection of blood and liver tissue at 4, 6, 9 h post insult. Intact right lobe of the liver was fixed in 10% formalin overnight for the paraffin section (Xiang et al., 2012) and the rest of liver tissue was frozen in −80°C. Sera diluted 10 times were sent to the *Department of Laboratory Medicine of Ruijin hospital, Shanghai Jiao Tong University School of Medicine* for the test of alanine transaminase (ALT) or aspartate transaminase (AST).

The following methods are described in Supplementary Material S1: Western blotting; Real-time quantitative polymerase chain reaction (RT-qPCR); Hematoxylin & eosin (H&E) staining; BrdU staining; Determination of mouse serum interleukin 22 (IL-22); Determination of malondialdehyde (MDA) in mouse liver tissue; Determination of multi-inflammatory cytokines in mouse serum.

### Statistical Analysis

Mean ± standard error (SEM) was used to describe the distribution of the data. Means for continuous variables were compared using paired or unpaired Student t-test between the two groups when the data were normally distributed. A *p*-value less than 0.05 was considered statistically significant.

## Results

### hUCBMNCs Treatment Prolonged Survival Time in the DAMP Plus PAMP-Induced Liver Injury Mouse Model

**FIGURE 1:**
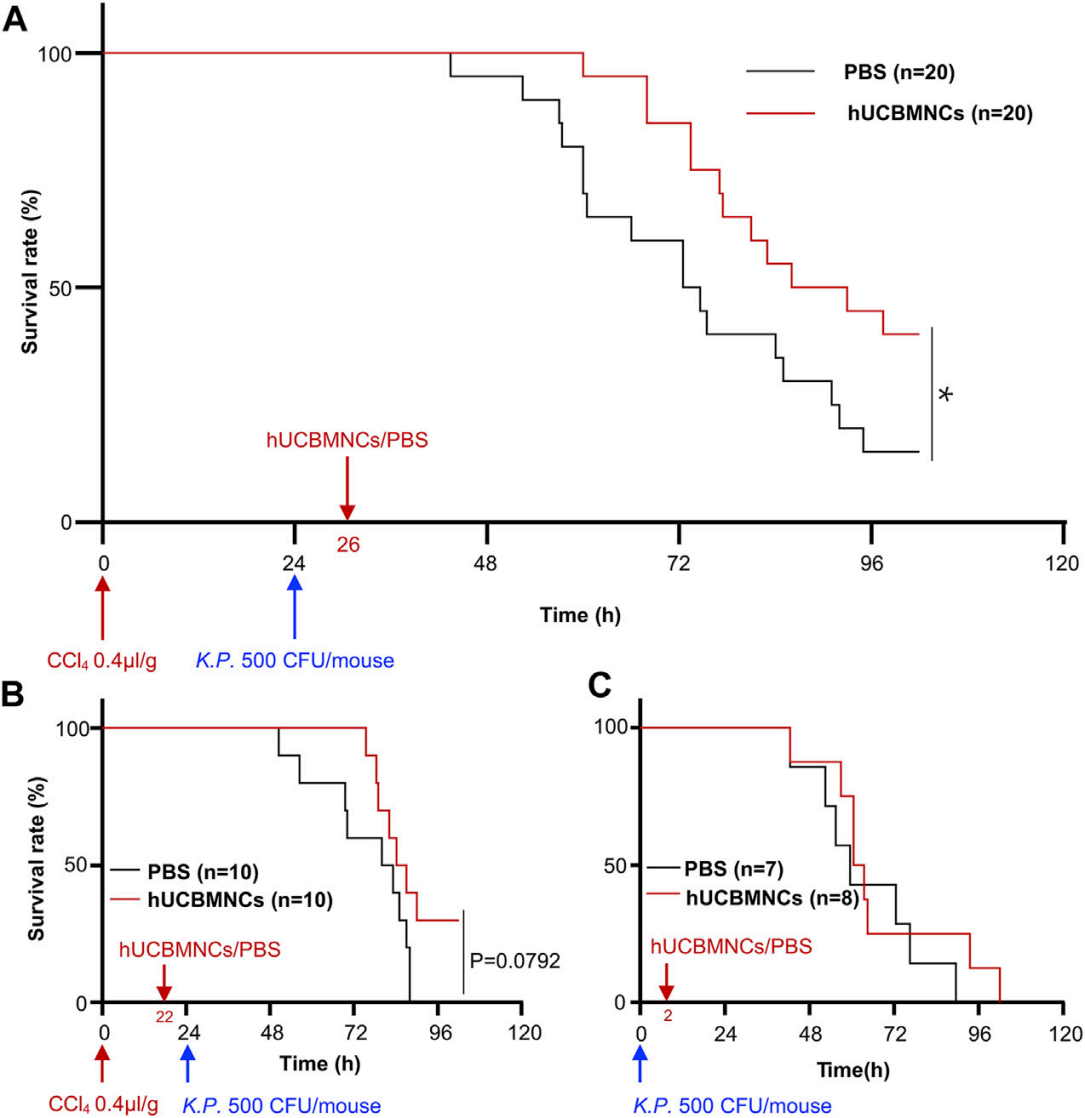
hUCBMNCs treatment prolonged mice survival time in DAMPs plus PAMPs-induced liver injury. **(A)** hUCBMNCs showed a therapeutic effect on CCl_4_ plus *Klebsiella pneumoniae* (*K.P.*) -induced liver injury. CCl_4_ (0.4 μl/g) plus *K.P.* (500 CFU/mouse) were administrated at 0 and 24h, respectively, to induce acute liver injury, and hUCBMNCs (*n* = 20) or PBS (*n* = 20) was applied at 26h for the observation of therapeutic effects. **(B)** Pretreatment of hUCBMNCs on CCl_4_ plus *K.P.*-induced liver injury hardly improved the mice survival. CCl_4_ (0.4 μl/g) plus *K.P.* (500 CFU/mouse) was administrated at 0 and 24h, respectively, to develop liver injury model, and hUCBMNCs (*n* = 10) or PBS (*n* = 10) was applied at 22h for the verification of preventative effects. **(C)** hUCBMNCs had no therapeutic effects on *K.P.*-induced liver injury. *K.P.* (500 CFU/mouse) was administrated intraperitoneally at 0h, and hUCBMNCs (*n* = 7) or PBS (*n* = 8) was applied at 2h post infection. *, *p* < 0.05.

Intravenous infusion of hUCBMNCs was applied 2 h before or after *K.P.* injection in CCl_4_ plus *K.P*.-induced acute liver injury mice. In the group of 2 h after *K.P.* injection, the application of hUCBMNCs showed an improved survival rate (*p* < 0.05) compared with the PBS group (Figure 1A). However, in the group of 2 h before *K.P.* injection, the survival rate showed no significant difference between the two groups (Figure 1B). To observe the exact function of hUCBMNCs in DAMP- or PAMP-induced inflammatory processes, we applied hUCBMNCs or PBS to PAMP-induced liver injury by using *K.P.* infection (Figure 1C) and found that there was no significant difference in survival rate between the two groups. hUCBMNCs treatment was also used in *S. Typhimurium* infection mouse model, and no significant difference was found as well (Supplementary Figure S2). These results indicated that hUCBMNCs might improve the damage of hepatocytes in DAMP-induced liver injury.

### hUCBMNCs Improved DAMP-Induced Acute Liver Injury in CCl_4_/ConA-Treated Mice

**FIGURE 2:**
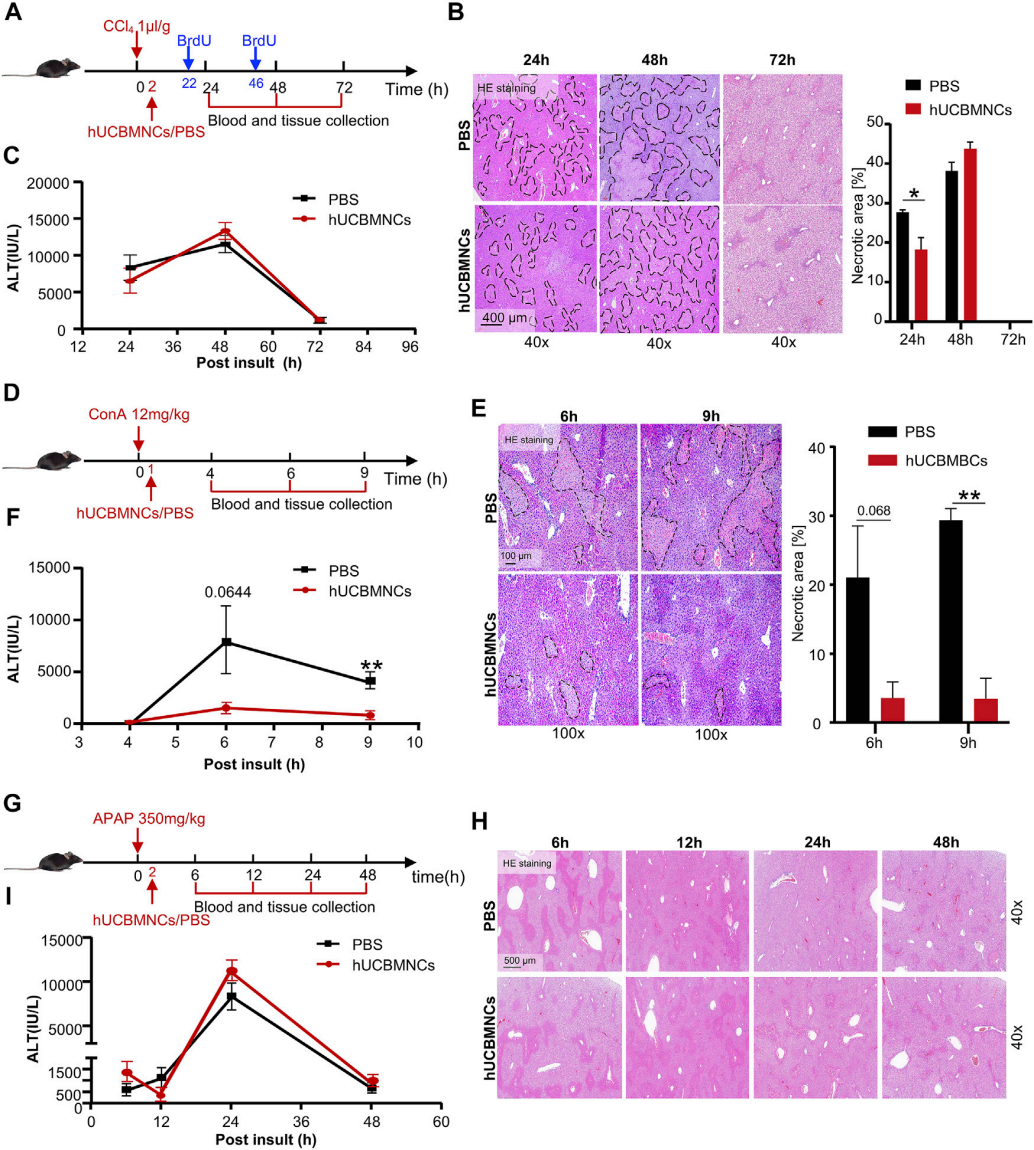
hUCBMNCs treatment improved CCl_4_/ConA-induced liver injury but did not improve APAP-induced liver injury. **(A–C)** hUCBMNCs treatment alleviated liver necrosis in CCl_4_-induced liver injury (PBS, *n* = 5/time point; hUCBMNCs, *n* = 5/time point). **(A)** Schematic experiment timeline of hUCBMNCs in CCl_4_-induced acute liver injury. **(B)** H&E staining (40×) and the percentage of necrotic areas of mice liver at 24, 48, and 72 h post CCl_4_ injection. Necrotic areas are circled with black dashed lines. **(C)** Serum ALT levels of mice at 24, 48, and 72 h post CCl_4_ insult. The bars represent mean ± SEM. **(D–F)** hUCBMNCs treatment alleviated liver necrosis in ConA-induced liver injury (PBS, *n* = 4/time point; hUCBMNCs, *n* = 4/time point). **(D)** Schematic experiment timeline of hUCBMNCs in ConA-induced acute liver injury. **(E)** H&E staining (100×) and the percentage of necrotic area of mice liver at 6 h, 9 h post ConA injection. Necrotic areas were circled with black dashed lines. **(F)** Serum ALT levels of mice at 4, 6, and 9 h post ConA injection. **(G–I)** hUCBMNCs treatment did not alleviate liver necrosis in APAP-induced liver injury (PBS, *n* = 4/time point; hUCBMNCs, *n* = 4/time point). **(G)** Schematic experiment timeline of hUCBMNCs in APAP-induced liver injury. **(H)** H&E staining (40×) of mice liver at 6, 12, 24, 48 h post APAP injection. **(I)** Serum ALT levels of mice at 6, 12, 24, and 48 h post APAP injection.

To verify the hypothesis that hUCBMNCs played a protective role in DAMP-induced liver injury, three kinds of acute liver injury mouse models induced by DAMPs were developed and hUCBMNCs were infused intravenously (Figures 2A,D,G). hUCBMNCs dramatically improved liver damages in CCl_4_-induced liver injury mouse model (Figure 2B) and ConA-induced liver injury mouse model (Figures 2E,F). However, hUCBMNCs did not alleviate liver damage in APAP-induced liver injury mouse model (Figures 2H,I). For CCl_4_-induced liver injury, although ALT levels showed no significant changes at the appointed time points, there was a significant difference in necrotic areas of liver H&E staining images at 24 h between the two groups post CCl_4_ injection (Figure 2B). H&E staining demonstrated that necrotic areas and degrees of necrosis were both ameliorated in the hUCBMNCs-treated group compared to the control group at 24h post CCl_4_ insult (Figure 2B). ALT levels, as a regular marker for assessing the severity of liver damage, were not significantly improved at 24, 48, and 72h post CCl_4_ insult (Figure 2C).

In ConA-induced acute liver injury, hUCBMNCs treatment significantly downregulated the necrotic areas [%] (Figure 2E) and ALT levels at 9h post insult (*p* < 0.01) were decreased correspondingly (Figure 2F). However, hUCBMNCs treatment did not improve APAP-induced acute liver injury significantly. The levels of liver necrosis areas (Figure 2H) and serum ALT (Figure 2I) in the APAP model were not improved after the treatment of hUCBMNCs, as well as mRNA levels of inflammatory cytokines (Supplementary Figure S3A). In addition, the phosphorylation levels of regeneration-associated phosphorylation of STAT3 (p-STAT3) protein were not enhanced in the liver but phosphorylation of ERK (p-ERK) was activated at 24h in the hUCBMNCs group after APAP insult (Supplementary Figure S3B).

### hUCBMNCs Alleviated Liver Inflammation and Oxidative Stress in CCl_4_/ConA-Induced Acute Liver Injury

Since inflammatory response and oxidative stress were two main pathological processes contributing to the deterioration of liver injury (Reyes-Gordillo et al., 2017), we assumed that hUCBMNCs treatment would attenuate the levels of inflammation and oxidative stress in both CCl_4_- and ConA-induced liver injury.

**FIGURE 3:**
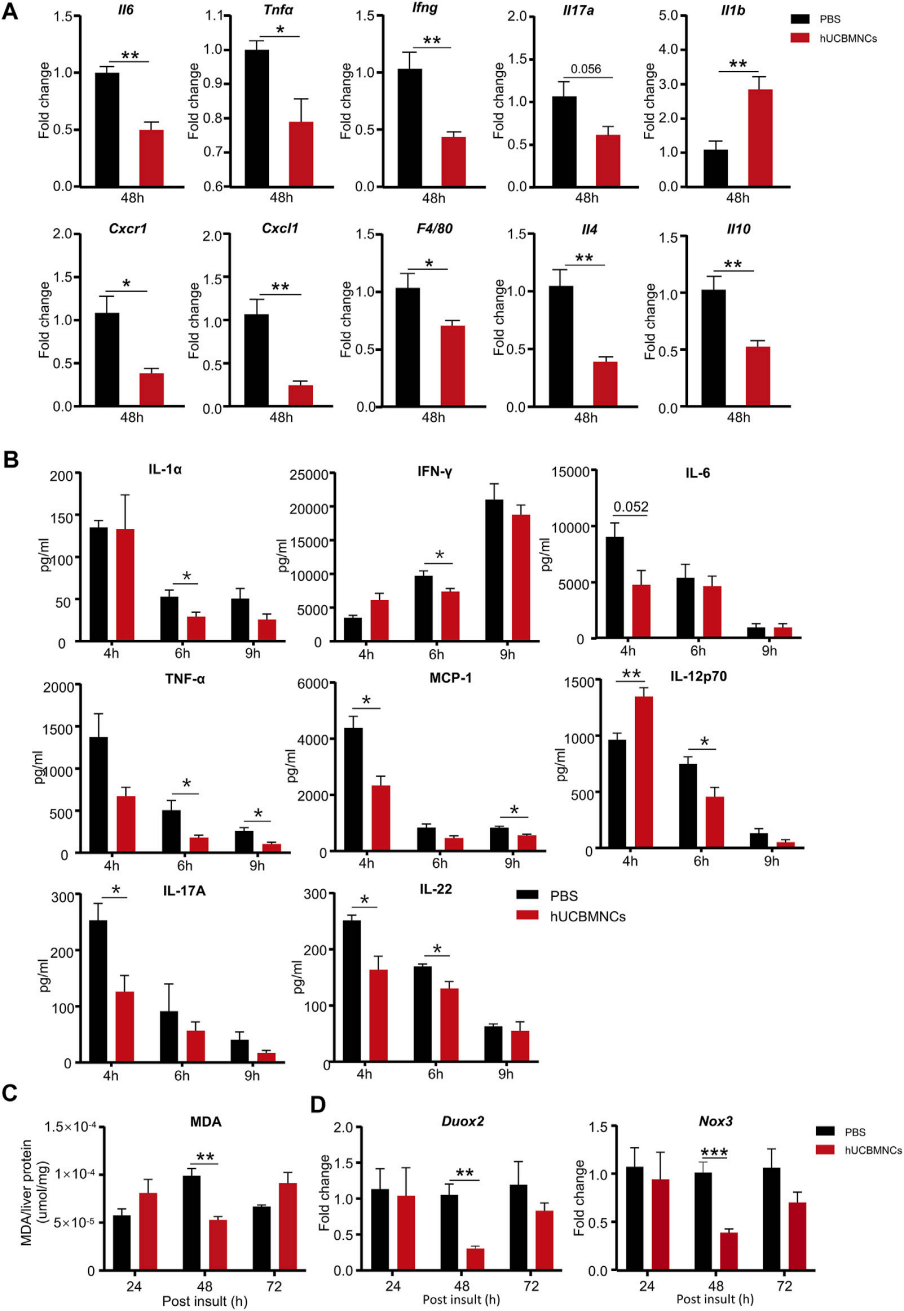
hUCBMNCs treatment suppressed inflammation and oxidative stress in mice liver in CCl_4_-induced acute liver injury and ConA-induced liver injury. **(A)** mRNA levels of liver representative inflammatory markers in hUCBMNCs and control groups at 48h post CCl_4_ injection (*n* = 5/time point, experiments were repeated for 3 times). **(B)** Protein levels of serum inflammatory cytokines in ConA-induced liver injury mouse model at 4, 6, and 9h (*n* = 5/time point). **(C)** Malondialdehyde (MDA) expression in liver tissue at 24, 48,and 72h post CCl_4_ insult (*n* = 5/time point, experiments were repeated for 3 times). **(D)** mRNA expression levels of *Duox2* and *Nox3* in liver at 24, 48, and 72h post CCl_4_ injection (*n* = 5/time point, experiments were repeated for 3 times). The bars represent mean ± SEM.

To confirm this hypothesis, hepatic mRNA levels of inflammatory markers were measured by RT-qPCR. hUCBMNCs treatment significantly suppressed mRNA expression of pro-inflammatory cytokines of *II6*, *Tnfα*, *Ifng*, *II17a* except for *II1b* in CCl_4_-induced liver injury at 48 h (Figure 3A). Moreover, the expression of *Cxcl1* and *Cxcr1* markedly declined after hUCBMNCs treatment at 48h (Figure 3A), but the expression levels of inflammatory markers were not decreased at 24 h (Supplementary Figure S4). *F4/80*, a mouse-specific macrophage marker, was also downregulated at the mRNA level compared with the control group at 48 h. Interestingly, mRNA levels of anti-inflammatory cytokines IL-4 and IL-10 were also decreased after the treatment of hUCBMNCs. Although hUCBMNCs treatment did not influence mRNA expression of inflammatory cytokines in ConA-induced liver injury (Supplementary Figure S5), protein levels of inflammatory cytokines such as TNF-α, IFN-γ, IL-17A, IL-1α, MCP-1, IL-12p70, and IL-22 were downregulated in our experiment (Figure 3B).

Subsequently, we determined the levels of oxidative stress in mice liver. The expression of MDA in liver homogenate supernatants was measured by MDA Assay Kit, and the levels of MDA were significantly decreased in the hUCBMNCs treatment group at 48h post CCl_4_ insult (*p* < 0.01) (Figure 3C). We further detected the expression of ROS-related oxidant enzymes and found that mRNA expression levels of NADPH oxidase Duox2 and Nox3 were decreased at 48h post CCl_4_ insult in the hUCBMNCs treatment group (Figure 3D).

### hUCBMNCs Played a Protective Role Against CCl_4_-Induced Acute Liver Injury via Upregulating IL-22

Improved necrosis of hepatocytes was demonstrated in hUCBMNCs-treated mice with CCl_4_-induced liver injury and ConA-induced liver injury. Whether liver regeneration was enhanced in these mice required further confirmation.

**FIGURE 4:**
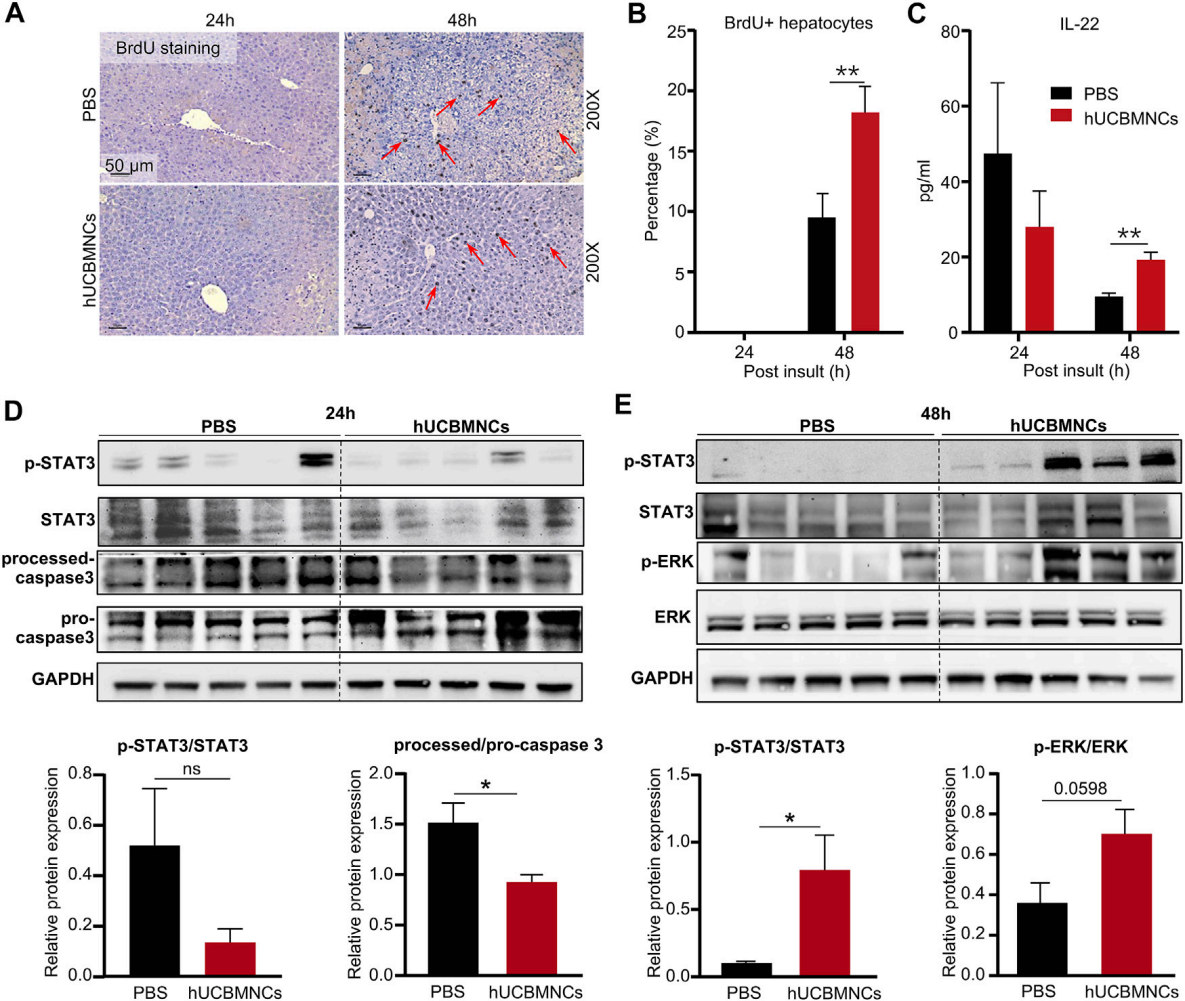
hUCBMNCs treatment promoted liver regeneration at 48h post CCl_4_ injection via IL-22. **(A)** BrdU staining of liver slices at 24 h, 48 h post CCl_4_ injection (PBS, *n* = 5/time point; hUCBMNCs, *n* = 5/time point). **(B)** Percentage of BrdU positive hepatocytes. **(C)** Serum IL-22 levels in the hUCBMNCs group (*n* = 4/time point) and the control group (*n* = 4/time point). **(D,E)** Western blotting results of proteins and relative protein expression in liver regeneration and apoptosis pathways, including p-STAT3, STAT3, processed-caspase 3, pro-caspase 3, p-ERK, ERK, and GAPDH at 24 h **(D)** and 48 h **(E)** post CCl_4_ injection (*n* = 5/time point, experiments were repeated for 3 times). The bars represent mean ± SEM.

In CCl_4_-induced mouse liver injury model, BrdU staining was performed on liver slices in mice. The percentage of BrdU-positive (BrdU+) hepatocytes was counted per ×200 field. Notably, hUCBMNCs treatment enhanced liver regeneration at 48 h post CCl_4_ injection. BrdU + hepatocytes were increased in the hUCBMNCs group compared to the control group at 48 h (*p* < 0.01) (Figures 4A,B). Subsequently, Western blotting results showed that the phosphorylation of STAT3 (p-STAT3) was strongly activated in the hUCBMNCs group at 48 h (*p* < 0.05) (Figure 4E), but the expression levels of phosphorylation of STAT1 (p-STAT1) at 24 and 48 h were not significantly different between the PBS treatment group and the hUCBMNCs treatment group (Supplementary Figure S6). Marginal activation of p-ERK protein in the hUCBMNCs group was also found compared with the control group at 48 h (Figure 4E). In ConA-induced mouse liver injury model, there were no significant differences of p-STAT1 and p-STAT3 at 6 and 9 h between the PBS treatment group and the hUCBMNCs treatment group (Supplementary Figure S7). IL-6 and IL-22 are both described as pro-proliferative cytokines in liver regeneration and the two cytokines signal through activation of STAT3 pathway (Schmidt-Arras and Rose-John, 2016; Xiang et al., 2020). The mRNA levels of IL-6 in liver were significantly decreased under the treatment of hUCBMNCs (Figure 3A). Thus, serum IL-22 levels were determined by using ELISA. Compared to PBS group, serum IL-22 levels were elevated under the treatment of hUCBMNCs at 48h. Usually, in normal mice, serum IL-22 is maintained at a low level (∼10 pg/ml) (Xu et al., 2014). After CCl_4_ injury, serum IL-22 levels were elevated both in PBS (∼40 pg/ml) and hUCBMNCs (∼30 pg/ml) groups at 24h. However, IL-22 levels declined to baseline (∼10 pg/ml) at 48h in the PBS group (Figure 4C). Interestingly, in the hUCBMNCs group, serum IL-22 levels were upregulated and maintained around 20 pg/ml (*p* < 0.001) at 48h (Figure 4C). Activation of caspase-3 protein was attenuated at 24 h under hUCBMNCs treatment (Figure 4D). But there was no significant difference in hepatocyte apoptosis at 48 and 72 h between the two groups via TUNEL staining (Supplementary Figure S8).

### hUCBMNCs Treatment Enhanced Autophagy of Hepatocyte and Promoted Liver Repair

Autophagy is widely involved in many pathophysiological courses, especially in oxidative stress and inflammation (You et al., 2015). We have previously found that IL-22 could protect drug-induced liver injury by enhancing autophagy (Lai et al., 2015; Mo et al., 2018). Therefore, we further explored whether enhanced autophagy would contribute to the protective effects of hUCBMNCs against CCl_4_-induced liver injury.

**FIGURE 5:**
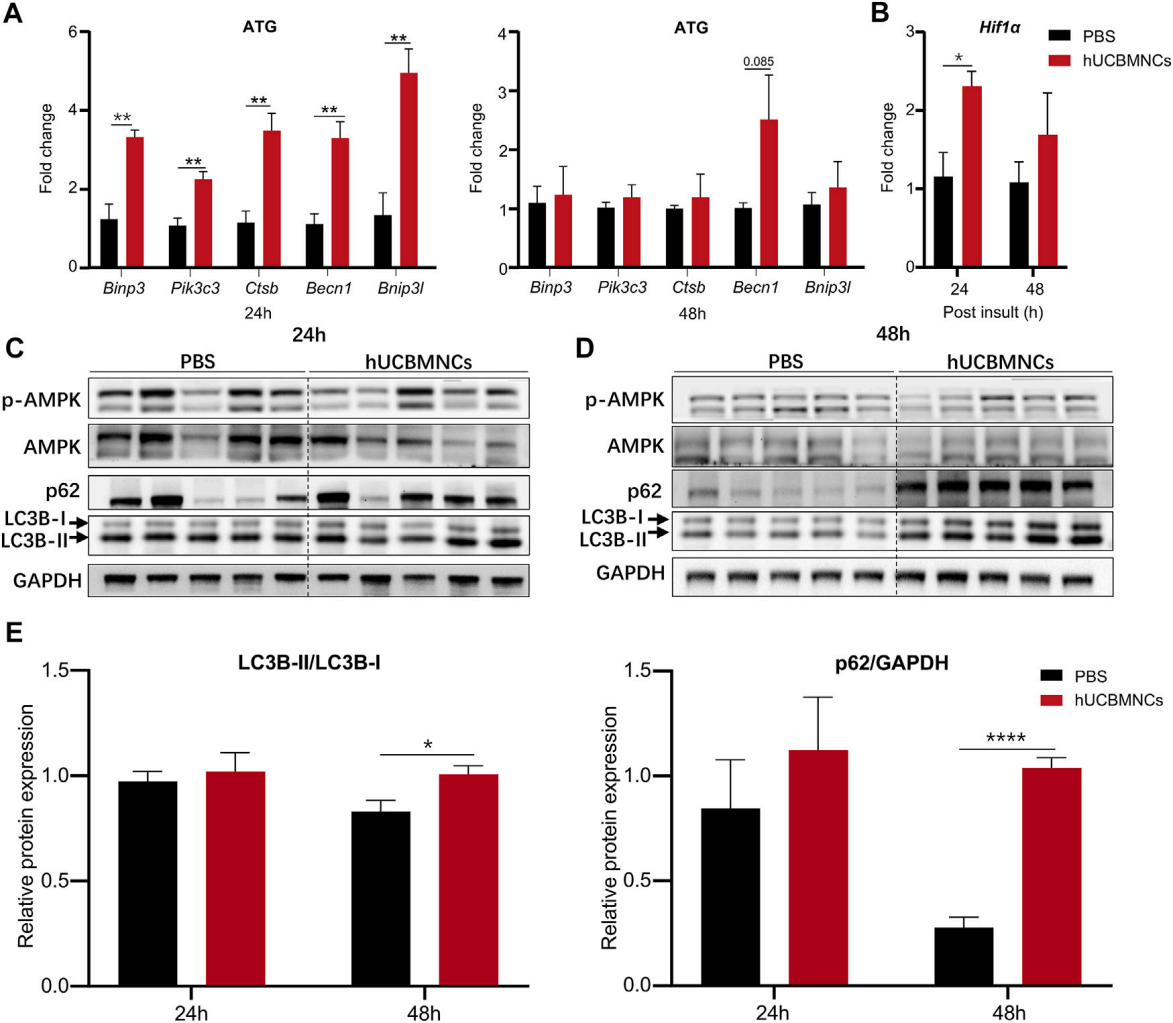
hUCBMNCs treatment enhanced hepatocellular autophagy at mRNA and protein levels. **(A)** mRNA levels of autophagy-related genes (ATG) in liver at 24 and 48h post CCl_4_ injection (*n* = 5/time point, experiments were repeated for 3 times). **(B)** Liver mRNA level of *hypoxia inducible factor 1α* (*Hif1α*) (*n* = 5/time point, experiments were repeated for 3 times). **(C,D)** Western blotting results of proteins in autophagy pathway in the liver, including p-AMPK, AMPK, p62, LC3B-I and LC3B-II at 24h **(C)** and 48h **(D)** post CCl_4_ injection (*n* = 5/time point, experiments were repeated for 3 times). **(E)** Relative protein expression of LC3B-II/LC3B-I, p62/GAPDH at 24 and 48 h post CCl_4_ insult (*n* = 5/time point, experiments were repeated for 3 times). The bars represent mean ± SEM.

A series of autophagy-related genes (ATG) regulate and participate in the process of autophagy. To confirm the involvement of autophagy in hUCBMNCs treatment in CCl_4_-induced liver injury, expression of ATG in the liver was detected by RT-qPCR. mRNA expression levels of *Binp3*, *Pik3c3*, *Ctsb*, *Becn1*, *Bnip3l* were significantly elevated at 24 h in the hUCBMNCs group compared with the control group (*p* < 0.01) (Figure 5A). Additionally, hUCBMNCs treatment induced enhanced expression of hypoxia-inducible factor 1α (Hif1α) (*p* < 0.05) (Figure 5B), which could interact with BINP3 to promote autophagy and exert beneficial effects on liver repair after damage (Zhang et al., 2019). Further assessment of autophagy markers was performed by Western blotting (Figures 5C,D), and expression levels of LC3B-II and p62 were significantly increased under hUCBMNCs treatment at 48 h (Figure 5E).

### Blocking IL-22 Could Partly Impair the Therapeutic Effects of hUCBMNCs

**FIGURE 6:**
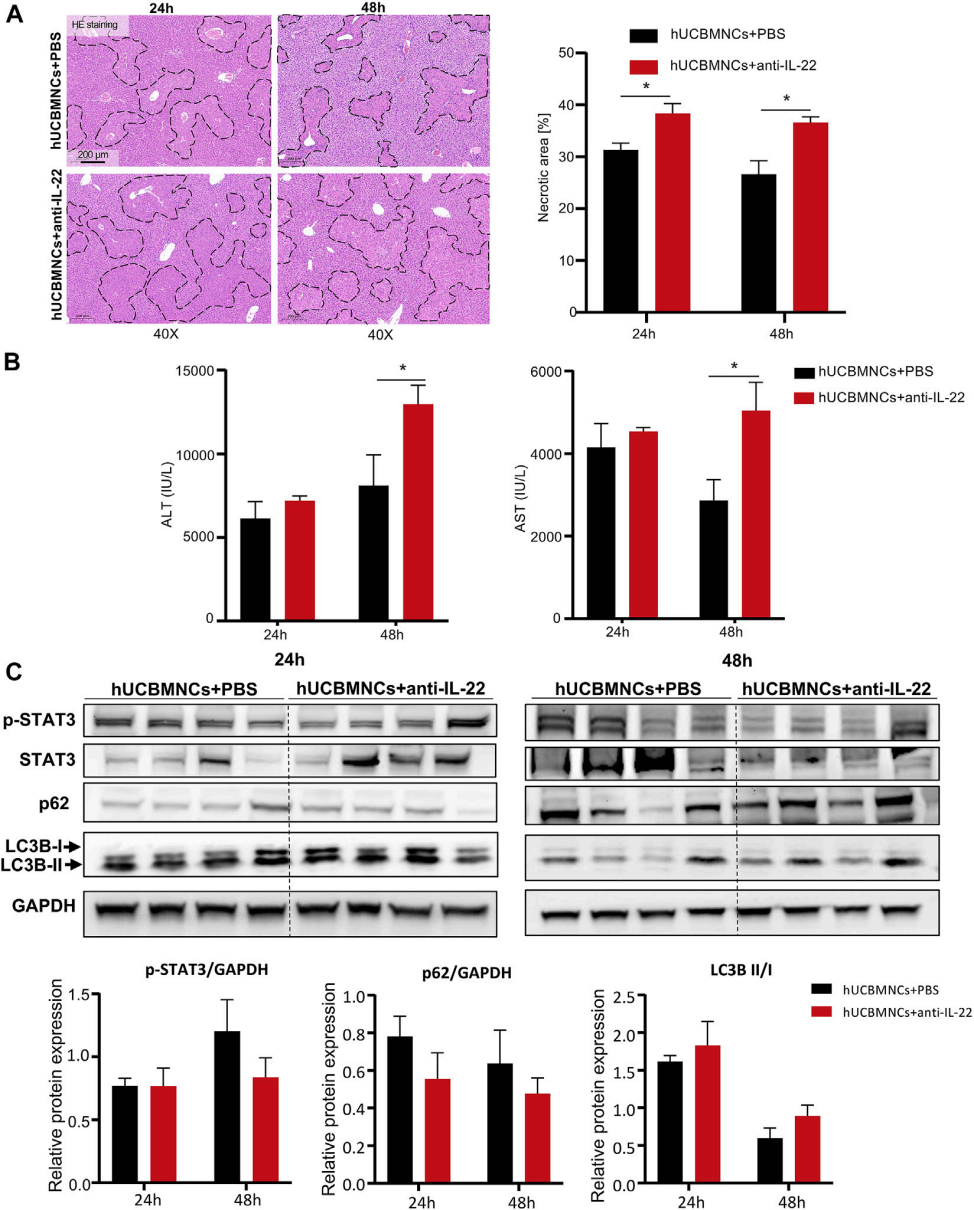
Blocking IL-22 impaired the therapeutic effects of hUCBMNCs. **(A)**. H&E staining (×40) and necrotic areas percentage of mice liver at 24 h, 48 h post CCl_4_ injection in the anti-IL-22 group and the control group (4 mice each group). **(B)** Serum ALT and AST levels of mice at 24 h, 48 h post CCl_4_ injection in the anti-IL-22 group and the control group (4 mice each group). **(C)** Expression of p-STAT3, p62, LC3B, and GAPDH of mice liver at 24 h, 48 h post CCl_4_ injection in the anti-IL-22 group and the control group (4 mice each group, experiments were repeated for 3 times). The bars represent mean ± SEM.

To confirm whether the upregulated peripheral IL-22 would contribute to the promoted liver regeneration and enhanced autophagy after hUCBMNCs treatment, we blocked peripheral IL-22 by administrating neutralizing IL-22 antibody synchronously. Liver H&E staining suggested that IL-22 neutralizing antibody impaired the therapeutic effects of hUCBMNCs (Figure 6A).

Serum ALT and AST levels were higher in the anti-IL-22 treatment group than that in the control group at 48h (Figure 6B). In addition, the expression levels of regeneration-related protein p-STAT3 were declined at 48h in anti-IL-22 mice (Figure 6C). The expression levels of autophagy-related protein p62 were also downregulated at both 24 and 48 hours after treating with anti-IL-22 antibody (Figure 6C). There were no significant differences of LC3B-II/LC3B-I (Figure 6C).

## Discussion

**FIGURE 7:**
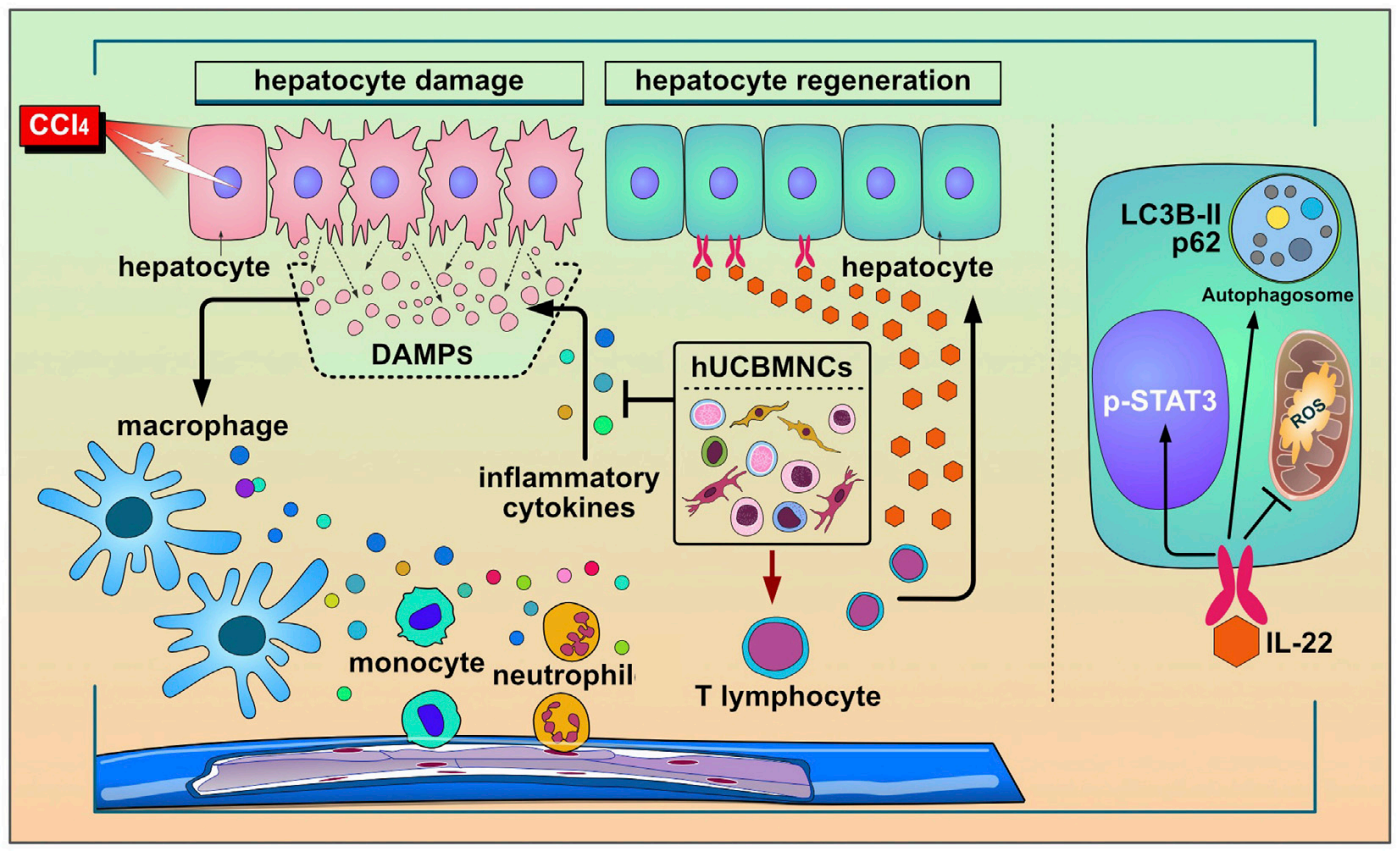
The working model for the protective roles of hUCBMNCs in acute liver injury/failure. hUCBMNCs treatment as a kind of cell therapeutic strategy could attenuate CCl_4_-induced acute liver injury/failure in mice with hepatoprotective role, which is executed by enhancing autophagy and regeneration in the liver via inhibiting inflammatory responses and upregulating peripheral IL-22.

To our knowledge, the present study is the first investigation to utilize hUCBMNCs to treat acute liver injury in mouse models induced by DAMPs or PAMPs. The hepato-protective role of hUCBMNCs was surprisingly found in CCl_4_/ConA-induced acute liver injury model. We have provided the first evidence that hUCBMNCs could alleviate DAMPs-induced liver injury including CCl_4_-/ConA- except APAP-induced liver injury. The hepatoprotective effects of hUCBMNCs were demonstrated as intensified pro-regeneration and enhanced autophagy in the liver, which turned out to be partly via upregulation of peripheral IL-22 level (Figure 7).

Cell therapies provide unprecedented opportunities for intractable diseases and injury (Yamanaka, 2020). The combined disciplines of cell and gene therapy and tissue engineering, broadly known as regenerative medicine, have the potential to revolutionize the treatment of diseases (De Luca et al., 2019). Mesenchymal stem cells (MSCs) have been the main subject of clinical trials for the functions of immunoregulation (Tasso and Pennesi, 2009), differentiation potential (Musina et al., 2006), pro-regeneration (Jackson et al., 2010), and anti-infective property (Shaw et al., 2021). Both hUCBMNCs and MSCs can be isolated from cord blood. hUCBMNCs have already presented with immunoregulation function in a variety of diseases (Alvarez-Mercado et al., 2008; Paton et al., 2019; Feng et al., 2020; Li et al., 2020). In the present study, hUCBMNCs treatment was revealed to play a protective role by improving survival rate in DAMPs plus PAMPs-induced liver injury mice. However, in PAMPs-induced liver injury mice, hUCBMNCs showed no protective effects on mouse models with bacterial infection including *K.P.* or *S. Typhimurium*, which indicated that hUCBMNCs treatment might have beneficial effects on DAMP-induced liver injury. Cell therapy as an emerging therapeutic strategy has been applied for many intractable liver diseases, such as acute liver injury, acute-on-chronic liver failure (Dwyer et al., 2021), and diabetic hepatocyte damage in mice (Nagaishi et al., 2014). It is reported that cells could successfully reach the liver after administrating intravenously in mice (Starkey Lewis et al., 2020). Several studies have shown that labeled cells can migrate to the injured liver by APAP and alleviate liver injury (Resaz et al., 2011; Nagaishi et al., 2014; Starkey Lewis et al., 2020). For hUCBMNCs treatment, we have no evidence showing that these cells would surely migrate to the liver in the present study, and we will perform this in the further study with cell labeling and tracking techniques.

CCl_4_- or APAP-induced acute liver injury mouse model is the representative animal model of hepatotoxin-mediated acute liver injury (Forbes and Newsome, 2016). The core pathological processes in both models are oxidative stress and inflammatory response (Reyes-Gordillo et al., 2017). In our study, hUCBMNCs treatment exerted hepatoprotective effects on CCl_4_-induced acute liver injury mice but could hardly improve the necrosis in APAP-induced mice, which is consistent with the effects of MSCs on APAP mice model (Ryu et al., 2014). Nonetheless, hUCBMNCs treatment indeed reduced the expression of p-ERK activation in the pathogenesis of APAP-induced liver damage. This indicated that hUCBMNCs could alleviate APAP-induced liver injury at the protein level. For ALT levels, though there were no statistically significant differences between the hUCBMNCs treatment group and the control group in CCl_4_- or APAP-induced liver injury mouse model, the elevated ALT levels could demonstrate the liver damage in the two models. In CCl_4_-induced liver injury model, ALT levels were elevated at 24 h and reached a peak level at 48 h, which indicated that liver damage already occurred at 24 h (Liu et al., 2015). However, in APAP-induced liver injury, ALT levels were elevated at 6h in our experiment. In fact, it was reported that serum ALT levels were elevated at 0.5 h and reached the peak at 24h in APAP-induced liver injury (Yi et al., 2021). Therefore, hepatic necrosis can be found at 6 h in APAP-induced liver injury. However, calculations based on the total amount of ALT in the damaged liver indicate that the increase in the serum ALT activity cannot be explained simply by leakage from necrotic cells (Hauss and Leppelmann, 1958; Freidman and Lapan, 1964). Poor correlation between the degree of necrosis and the magnitude of serum ALT levels has often been reported (Rees and Sinha, 1960). Thus, hepatic histopathologic changes are always needed as the gold standard to confirm liver necrosis (Khalifa and Rockey, 2020). In liver H&E staining, the necrotic areas of mice liver at 24h post CCl_4_ injection were significantly decreased in the hUCBMNCs treatment group, though there was no significant difference of ALT levels between the two groups.

ConA-induced T-cell-mediated liver injury is a well-established model for investigating autoimmune or viral fulminant hepatitis with acute immune response playing a pivotal role in mediating liver damage (Wang et al., 2012). Immunosuppression therapy has an obvious effect on autoimmune hepatitis (Mieli-Vergani et al., 2018). In the present study, hUCBMNCs treatment demonstrated a dramatic attenuation of ConA-induced liver injury. Unlike hUCBMNCs in CCl_4_-induced liver injury, improved liver function and necrosis were both shown in ConA-induced liver injury. A previous study demonstrated that glucocorticoids, as immunosuppressants, have opposing effects on CCl_4_-induced liver injury and ConA-induced liver injury (Kwon et al., 2014). hUCBMNCs, reported for the immunomodulatory functions, showed hepatoprotective effects both on CCl_4_- and ConA-induced acute liver injury in the present study. Therefore, hUCBMNCs treatment would provide an alternative therapeutic strategy in clinical practice. The underlying mechanism of hUCBMNCs on ConA-induced liver injury needs to be further explored.

Our study showed that hUCBMNCs alleviated the levels of inflammatory responses and oxidative stress in the liver at 48 h post-CCl_4_ injection. The process of oxidative stress induces cells death involved a wide range of DAMPs (Jaeschke, 2011; Yang and Tonnesseen, 2019), and then activates innate immune cells to release abundant of inflammatory cytokines. The aggravation of inflammatory response would contribute to systemic inflammatory response syndrome (SIRS), and eventually lead to multiple organ failures (Rolando et al., 2000). Our study revealed that hUCBMNCs treatment alleviated both inflammatory responses (*II6*, *Tnfa*, *Ifng*, *II17a*, *II1b*, *Cxcl1*, *Cxcr1*, *F4/80*) and oxidative stress in CCl_4_-induced liver injury. Further experiments uncovered that NADPH oxidase *Duox2* and *Nox3*, leading to accumulation of peroxides (Babior, 2004), were also downregulated at mRNA levels at 48h in the hUCBMNCs group. These results demonstrated the promising and inspiring therapeutic values for acute liver injury of hUCBMNCs treatment.

Besides, it was exciting that hUCBMNCs enhanced the phosphorylation levels of STAT3 protein, which would promote liver regeneration in CCl_4_-induced acute liver injury (Gao, 2005). And BrdU staining also confirmed the effect of pro-regeneration in hUCBMNCs treated mice at 48 h. IL-6 is a critical pro-regenerative factor and acute-phase inducer. IL-6 activates downstream signal through p-STAT3 in the liver, which confers resistance to liver damage (Schmidt-Arras and Rose-John, 2016). However, it was reported that mRNA and protein levels of IL-6 were both inhibited after treatment of hUCBMNCs (Rosenkranz et al., 2012; Yu et al., 2021), which are parallel to our study. The inhibition of IL-6 expression by hUCBMNCs in the early stage of liver injury would theoretically lead to the inhibition of liver regeneration which might be harmful for liver repair. However, the elevated levels of serum IL-22 could partly illustrate the activation of p-STAT3 in the liver. In addition, the attenuated phosphorylation levels of STAT3 (p-STAT3) after blocking peripheral IL-22 could partly demonstrate the pro-regenerative effect of hUCBMNCs. IL-22, a member of the IL-10 family, is mainly produced by innate lymphoid cells, Th17 cells, and Th22 cells (Radaeva et al., 2004; Xiang et al., 2012; Dudakov et al., 2015; Gao and Xiang, 2019). The function of IL-22 is mainly mediated via the activation of the Jak1/Tyk2-STAT3 pathway by binding to the heterodimeric receptors of IL-22R1 and IL-10R2 (Kong et al., 2013). IL-22 was mainly reported for its robust effects of pro-regenerative and hepatoprotective roles (Pan et al., 2004; Radaeva et al., 2004). Interestingly, compared to PBS treatment, hUCBMNCs treatment upregulated serum IL-22 in CCl_4_-induced liver injury mouse model, but downregulated serum IL-22 in ConA-induced liver injury mouse model. Considering that serum IL-22 was reduced to ∼10 pg/ml in the PBS treatment group, mildly elevated serum IL-22 in CCl_4_-induced liver injury mice might possibly be secreted by hUCBMNCs. It was reported that IL-22 was mainly produced by lymphoid cells (Dudakov et al., 2015). Our flow cytometry results of the constituents of hUCBMNCs manifested that there were activated T cells in hUCBMNCs, including NK cells and CD4^+^ T cells. These cells could secret IL-22, which would contribute to the increased serum level of IL-22 and the promoted liver regeneration at 48 h (Xiang et al., 2020).

Autophagy is a crucial process for maintaining cellular homeostasis (Dunlop et al., 2014), and many genes are involved in autophagy regulation. We further explored the expression of autophagy-related genes (ATG) in mouse liver. The expression levels of *Binp3*, *Pik3c3*, *Ctsb*, *Becn1*, and *Bnip3l* were significantly elevated under the treatment of hUCBMNCs. Among these targets, BECN1 and PIK3C3 are involved in the initiation of autophagy (Feng et al., 2014). BECN1 is reported as a direct transcriptional target of STAT3, and the phosphorylation of STAT3 after IL-6 administration could inhibit BECN1 expression at mRNA and protein levels (Miao et al., 2014). But in the present study, hUCBMNCs treatment promoted the phosphorylation of STAT3 at 48 h and did not suppress mRNA expression of BECN1. Further investigation is still needed. BINP3 and BINP3L proteins are crucial for hypoxia-induced autophagy (Bellot et al., 2009). Elevated HIF-1α has a protective effect on hepatocytes in liver injury (Khan et al., 2017). hUCBMNCs treatment promoted the expression of HIF-1α. BNIP3, a HIF-1α interacting protein, which was known to play a fundamental role in response to hypoxia (Zhang et al., 2019), was also increased at the mRNA level after hUCBMNCs treatment. LC3B-II protein, the cleaved and lapidated form of autophagy protein LC3B, is a widely known marker of autophagy (Dikic and Elazar, 2018). In our study, the expression of LC3B-II and p62 was significantly enhanced. These elevated expression levels of autophagy-related genes and autophagy proteins strongly suggested that autophagy played a role in the hepatoprotective effects after hUCBMNCs treatment.

In the present study, hUCBMNCs showed various effects on CCl_4_-induced liver injury. In our opinion, hUCBMNCs treatment might alleviate CCl_4_-induced liver injury through the elevation of peripheral IL-22 (Figure 7). With low immunogenicity (Kang et al., 2020), hUCBMNCs are more convenient to obtain from cord blood compared to MSCs, and the functions of hUCBMNCs with anti-inflammation, pro-regeneration and enhancement of autophagy in CCl_4_-induced hepatotoxin-mediated liver injury highlight that hUCBMNCs could be an alternative optimal cell therapy strategy for patients with liver injury or liver failure.

## Data Availability

The raw data supporting the conclusions of this article will be made available by the authors, without undue reservation.

## References

[B1] Alvarez-Mercado A. I. Sáez-Lara M. J. García-Mediavilla M. V. Sánchez-Campos S. Abadía F. Cabello-Donayre M. (2008). Xenotransplantation of Human Umbilical Cord Blood Mononuclear Cells to Rats with D-Galactosamine-Induced Hepatitis. Cell Transpl. 17 (7), 845–857. 10.3727/096368908786516837 10.3727/096368908786516837 | Google Scholar 19044210

[B2] Arroyo V. Moreau R. Jalan R. (2020). Acute-on-Chronic Liver Failure. N. Engl. J. Med. 382 (22), 2137–2145. 10.1056/NEJMra1914900 PubMed Abstract | 10.1056/NEJMra1914900 | Google Scholar 32459924

[B3] Babior B. M. (2004). NADPH Oxidase. Curr. Opin. Immunol. 16 (1), 42–47. 10.1016/j.coi.2003.12.001 PubMed Abstract | 10.1016/j.coi.2003.12.001 | Google Scholar 14734109

[B4] Bellot G. Garcia-Medina R. Gounon P. Chiche J. Roux D. Pouysségur J. (2009). Hypoxia-Induced Autophagy Is Mediated through Hypoxia-Inducible Factor Induction of BNIP3 and BNIP3L via Their BH3 Domains. Mol. Cell Biol. 29 (10), 2570–2581. 10.1128/MCB.00166-09 PubMed Abstract | 10.1128/MCB.00166-09 | Google Scholar 19273585 PMC2682037

[B5] Bianchi M. E. (2007). DAMPs, PAMPs and Alarmins: All We Need to Know about Danger. J. Leukoc. Biol. 81 (1), 1–5. 10.1189/jlb.0306164 PubMed Abstract | 10.1189/jlb.0306164 | Google Scholar 17032697

[B6] Bird T. G. Müller M. Boulter L. Vincent D. F. Ridgway R. A. Lopez-Guadamillas E. (2018). TGFβ Inhibition Restores a Regenerative Response in Acute Liver Injury by Suppressing Paracrine Senescence. Sci. Transl. Med. 10, eaan1230. 10.1126/scitranslmed.aan1230 PubMed Abstract | 10.1126/scitranslmed.aan1230 | Google Scholar 30111642 PMC6420144

[B7] Bower W. A. Johns M. Margolis H. S. Williams I. T. Bell B. P. (2007). Population-based Surveillance for Acute Liver Failure. Am. J. Gastroenterol. 102 (11), 2459–2463. 10.1111/j.1572-0241.2007.01388.x PubMed Abstract | 10.1111/j.1572-0241.2007.01388.x | Google Scholar 17608778

[B8] Chew C. A. Iyer S. G. Kow A. W. C. Madhavan K. Wong A. S. T. Halazun K. J. (2020). An International Multicenter Study of Protocols for Liver Transplantation during a Pandemic: A Case for Quadripartite Equipoise. J. Hepatol. 73 (4), 873–881. 10.1016/j.jhep.2020.05.023 PubMed Abstract | 10.1016/j.jhep.2020.05.023 | Google Scholar 32454041 PMC7245234

[B9] Chung R. T. Stravitz R. T. Fontana R. J. Schiodt F. V. Mehal W. Z. Reddy K. R. (2012). Pathogenesis of Liver Injury in Acute Liver Failure. Gastroenterology 143 (3), e1–e7. 10.1053/j.gastro.2012.07.011 10.1053/j.gastro.2012.07.011 | Google Scholar PMC3641754 22796239

[B10] Dalous J. Pansiot J. Pham H. Chatel P. Nadaradja C. D'Agostino I. (2013). Use of Human Umbilical Cord Blood Mononuclear Cells to Prevent Perinatal Brain Injury: a Preclinical Study. Stem Cells Dev. 22 (1), 169–179. 10.1089/scd.2012.0183 PubMed Abstract | 10.1089/scd.2012.0183 | Google Scholar 22621245

[B11] De Luca M. Aiuti A. Cossu G. Parmar M. Pellegrini G. Robey P. G. (2019). Advances in Stem Cell Research and Therapeutic Development. Nat. Cell Biol. 21 (7), 801–811. 10.1038/s41556-019-0344-z PubMed Abstract | 10.1038/s41556-019-0344-z | Google Scholar 31209293

[B12] Dikic I. Elazar Z. (2018). Mechanism and Medical Implications of Mammalian Autophagy. Nat. Rev. Mol. Cell Biol. 19 (6), 349–364. 10.1038/s41580-018-0003-4 PubMed Abstract | 10.1038/s41580-018-0003-4 | Google Scholar 29618831

[B13] Dudakov J. A. Hanash A. M. van den Brink M. R. (2015). Interleukin-22: Immunobiology and Pathology. Annu. Rev. Immunol. 33, 747–785. 10.1146/annurev-immunol-032414-112123 PubMed Abstract | 10.1146/annurev-immunol-032414-112123 | Google Scholar 25706098 PMC4407497

[B14] Dunlop E. A. Seifan S. Claessens T. Behrends C. Kamps M. A. Rozycka E. (2014). FLCN, a Novel Autophagy Component, Interacts with GABARAP and Is Regulated by ULK1 Phosphorylation. Autophagy 10 (10), 1749–1760. 10.4161/auto.29640 PubMed Abstract | 10.4161/auto.29640 | Google Scholar 25126726 PMC4198360

[B15] Dwyer B. J. Macmillan M. T. Brennan P. N. Forbes S. J. (2021). Cell Therapy for Advanced Liver Diseases: Repair or Rebuild. J. Hepatol. 74 (1), 185–199. 10.1016/j.jhep.2020.09.014 PubMed Abstract | 10.1016/j.jhep.2020.09.014 | Google Scholar 32976865

[B16] Escorsell A. Mas A. de la Mata M. (2007). Acute Liver Failure in Spain: Analysis of 267 Cases. Liver Transpl. 13 (10), 1389–1395. 10.1002/lt.21119 PubMed Abstract | 10.1002/lt.21119 | Google Scholar 17370334

[B17] Feng Y. He D. Yao Z. Klionsky D. J. (2014). The Machinery of Macroautophagy. Cell Res. 24 (1), 24–41. 10.1038/cr.2013.168 PubMed Abstract | 10.1038/cr.2013.168 | Google Scholar 24366339 PMC3879710

[B18] Feng L. X. Zhao F. Liu Q. Peng J. C. Duan X. J. Yan P. (2020). Role of Nrf2 in Lipopolysaccharide-Induced Acute Kidney Injury: Protection by Human Umbilical Cord Blood Mononuclear Cells. Oxid. Med. Cell Longev. 2020, 6123459. 10.1155/2020/6123459 PubMed Abstract | 10.1155/2020/6123459 | Google Scholar 32774680 PMC7407026

[B19] Forbes S. J. Newsome P. N. (2016). Liver Regeneration - Mechanisms and Models to Clinical Application. Nat. Rev. Gastroenterol. Hepatol. 13 (8), 473–485. 10.1038/nrgastro.2016.97 PubMed Abstract | 10.1038/nrgastro.2016.97 | Google Scholar 27353402

[B20] Freidman M. M. Lapan B. (1964). Enzyme Activities During Hepatic Injury Caused by Carbon Tetrachloride. Clin. Chem. 10, 335–345. PubMed Abstract | Google Scholar 14165562

[B21] Gao B. Xiang X. (2019). Interleukin-22 from Bench to Bedside: a Promising Drug for Epithelial Repair. Cell Mol. Immunol. 16 (7), 666–667. 10.1038/s41423-018-0055-6 PubMed Abstract | 10.1038/s41423-018-0055-6 | Google Scholar 29921965 PMC6804818

[B22] Gao B. (2005). Cytokines, STATs and Liver Disease. Cell Mol. Immunol. 2 (2), 92–100. PubMed Abstract | Google Scholar 16191414

[B23] Hauss W. H. Leppelmann H. J. (1958). Reactive Changes of Enzyme Activities in Serum and Liver as Symptoms of Acute Syndrome. Ann. N. Y. Acad. Sci. 75 (1), 250–259. 10.1111/j.1749-6632.1958.tb36871.x PubMed Abstract | 10.1111/j.1749-6632.1958.tb36871.x | Google Scholar 13627823

[B24] He Y. Feng D. Hwang S. Mackowiak B. Wang X. Xiang X. (2021). Interleukin-20 Exacerbates Acute Hepatitis and Bacterial Infection by Downregulating IjBf Target Genes in Hepatocytes. J. Hepatol. 75, 163–176. 10.1016/j.jhep.2021.02.004 PubMed Abstract | 10.1016/j.jhep.2021.02.004 | Google Scholar 33610678 PMC8323118

[B25] Jackson W. M. Nesti L. J. Tuan R. S. (2010). Potential Therapeutic Applications of Muscle-Derived Mesenchymal Stem and Progenitor Cells. Expert Opin. Biol. Ther. 10 (4), 505–517. 10.1517/14712591003610606 PubMed Abstract | 10.1517/14712591003610606 | Google Scholar 20218920 PMC3018682

[B26] Jaeschke H. (2011). Reactive Oxygen and Mechanisms of Inflammatory Liver Injury: Present Concepts. J. Gastroenterol. Hepatol. 26 Suppl 1 (Suppl. 1), 173–179. 10.1111/j.1440-1746.2010.06592.x PubMed Abstract | 10.1111/j.1440-1746.2010.06592.x | Google Scholar 21199529

[B27] Ji Y. W. Mittal S. K. Hwang H. S. Chang E. J. Lee J. H. Seo Y. (2017). Lacrimal Gland-Derived IL-22 Regulates IL-17-mediated Ocular Mucosal Inflammation. Mucosal Immunol. 10 (5), 1202–1210. 10.1038/mi.2016.119 PubMed Abstract | 10.1038/mi.2016.119 | Google Scholar 28051088 PMC5496813

[B28] Kang J. Y. Oh M. K. Joo H. Park H. S. Chae D. H. Kim J. (2020). Xeno-Free Condition Enhances Therapeutic Functions of Human Wharton's Jelly-Derived Mesenchymal Stem Cells against Experimental Colitis by Upregulated Indoleamine 2,3-Dioxygenase Activity. J. Clin. Med. 9 (9), 2913. 10.3390/jcm9092913 PubMed Abstract | 10.3390/jcm9092913 | Google Scholar PMC7565923 32927587

[B29] Khalifa A. Rockey D. C. (2020). The Utility of Liver Biopsy in 2020. Curr. Opin. Gastroenterol. 36 (3), 184–191. 10.1097/mog.0000000000000621 PubMed Abstract | 10.1097/mog.0000000000000621 | Google Scholar 32097176 PMC10874678

[B30] Khan H. A. Ahmad M. Z. Khan J. A. Arshad M. I. (2017). Crosstalk of Liver Immune Cells and Cell Death Mechanisms in Different Murine Models of Liver Injury and its Clinical Relevance. Hepatobiliary Pancreat. Dis. Int. 16 (3), 245–256. 10.1016/s1499-3872(17)60014-6 PubMed Abstract | 10.1016/s1499-3872(17)60014-6 | Google Scholar 28603092 PMC7172563

[B31] Kong X. Feng D. Mathews S. Gao B. (2013). Hepatoprotective and Anti-fibrotic Functions of Interleukin-22: Therapeutic Potential for the Treatment of Alcoholic Liver Disease. J. Gastroenterol. Hepatol. 28 Suppl 1 (Suppl. 10 1), 56–60. 10.1111/jgh.12032 PubMed Abstract | 10.1111/jgh.12032 | Google Scholar 23855297 PMC3779467

[B32] Kwon H. J. Won Y. S. Park O. Feng D. Gao B. (2014). Opposing Effects of Prednisolone Treatment on T/NKT Cell- and Hepatotoxin-Mediated Hepatitis in Mice. Hepatology 59 (3), 1094–1106. 10.1002/hep.26748 PubMed Abstract | 10.1002/hep.26748 | Google Scholar 24115096 PMC3943761

[B33] Lai R. Xiang X. Mo R. Bao R. Wang P. Guo S. (2015). Protective Effect of Th22 Cells and Intrahepatic IL-22 in Drug Induced Hepatocellular Injury. J. Hepatol. 63 (1), 148–155. 10.1016/j.jhep.2015.02.004 PubMed Abstract | 10.1016/j.jhep.2015.02.004 | Google Scholar 25681556

[B34] Li X. W. Feng L. X. Zhu X. J. Liu Q. Wang H. S. Wu X. (2020). Human Umbilical Cord Blood Mononuclear Cells Protect against Renal Tubulointerstitial Fibrosis in Cisplatin-Treated Rats. Biomed. Pharmacother. 121, 109310. 10.1016/j.biopha.2019.109310 PubMed Abstract | 10.1016/j.biopha.2019.109310 | Google Scholar 31710895

[B35] Liu J. Zhang Q. Y. Yu L. M. Liu B. Li M. Y. Zhu R. Z. (2015). Phycocyanobilin Accelerates Liver Regeneration and Reduces Mortality Rate in Carbon Tetrachloride-Induced Liver Injury Mice. World J. Gastroenterol. 21 (18), 5465–5472. 10.3748/wjg.v21.i18.5465 PubMed Abstract | 10.3748/wjg.v21.i18.5465 | Google Scholar 25987768 PMC4427667

[B36] Miao L. J. Huang F. X. Sun Z. T. Zhang R. X. Huang S. F. Wang J. (2014). Stat3 Inhibits Beclin 1 Expression through Recruitment of HDAC3 in Nonsmall Cell Lung Cancer Cells. Tumour Biol. 35 (7), 7097–7103. 10.1007/s13277-014-1961-6 PubMed Abstract | 10.1007/s13277-014-1961-6 | Google Scholar 24760274

[B37] Mieli-Vergani G. Vergani D. Czaja A. J. Manns M. P. Krawitt E. L. Vierling J. M. (2018). Autoimmune Hepatitis. Nat. Rev. Dis. Prim. 4, 18017. 10.1038/nrdp.2018.17 PubMed Abstract | 10.1038/nrdp.2018.17 | Google Scholar 29644994

[B38] Mihm S. (2018). Danger-Associated Molecular Patterns (DAMPs): Molecular Triggers for Sterile Inflammation in the Liver. Int. J. Mol. Sci. 19 (10). 10.3390/ijms19103104 PubMed Abstract | 10.3390/ijms19103104 | Google Scholar PMC6213769 30309020

[B39] Mo R. Lai R. Lu J. Zhuang Y. Zhou T. Jiang S. (2018). Enhanced Autophagy Contributes to Protective Effects of IL-22 against Acetaminophen-Induced Liver Injury. Theranostics 8 (15), 4170–4180. 10.7150/thno.25798 PubMed Abstract | 10.7150/thno.25798 | Google Scholar 30128045 PMC6096391

[B40] Musina R. A. Bekchanova E. S. Belyavskii A. V. Sukhikh G. T. (2006). Differentiation Potential of Mesenchymal Stem Cells of Different Origin. Bull. Exp. Biol. Med. 141 (1), 147–151. 10.1007/s10517-006-0115-2 PubMed Abstract | 10.1007/s10517-006-0115-2 | Google Scholar 16929987

[B41] Nagaishi K. Ataka K. Echizen E. Arimura Y. Fujimiya M. (2014). Mesenchymal Stem Cell Therapy Ameliorates Diabetic Hepatocyte Damage in Mice by Inhibiting Infiltration of Bone Marrow-Derived Cells. Hepatology 59 (5), 1816–1829. 10.1002/hep.26975 PubMed Abstract | 10.1002/hep.26975 | Google Scholar 24375439

[B42] Pan H. Hong F. Radaeva S. Gao B. (2004). Hydrodynamic Gene Delivery of Interleukin-22 Protects the Mouse Liver from Concanavalin A-, Carbon Tetrachloride-, and Fas Ligand-Induced Injury via Activation of STAT3. Cell Mol. Immunol. 1 (1), 43–49. PubMed Abstract | Google Scholar 16212920

[B43] Paton M. C. B. Allison B. J. Fahey M. C. Li J. Sutherland A. E. Pham Y. (2019). Umbilical Cord Blood versus Mesenchymal Stem Cells for Inflammation-Induced Preterm Brain Injury in Fetal Sheep. Pediatr. Res. 86 (2), 165–173. 10.1038/s41390-019-0366-z PubMed Abstract | 10.1038/s41390-019-0366-z | Google Scholar 30858474

[B44] Radaeva S. Sun R. Pan H. N. Hong F. Gao B. (2004). Interleukin 22 (IL-22) Plays a Protective Role in T Cell-Mediated Murine Hepatitis: IL-22 Is a Survival Factor for Hepatocytes via STAT3 Activation. Hepatology 39 (5), 1332–1342. 10.1002/hep.20184 PubMed Abstract | 10.1002/hep.20184 | Google Scholar 15122762

[B45] Rees K. R. Sinha K. P. (1960). Blood Enzymes in Liver Injury. J. Pathol. Bacteriol. 80, 297–307. 10.1002/path.1700800213 PubMed Abstract | 10.1002/path.1700800213 | Google Scholar 13740296

[B46] Ren J. Sang Y. Qin R. Su Y. Cui Z. Mang Z. (2019). Metabolic Intermediate Acetyl Phosphate Modulates Bacterial Virulence via Acetylation. Emerg. Microbes Infect. 8 (1), 55–69. 10.1080/22221751.2018.1558963 PubMed Abstract | 10.1080/22221751.2018.1558963 | Google Scholar 30866760 PMC6455138

[B47] Resaz R. Emionite L. Vanni C. Astigiano S. Puppo M. Lavieri R. (2011). Treatment of Newborn G6pc(-/-) Mice with Bone Marrow-Derived Myelomonocytes Induces Liver Repair. J. Hepatol. 55 (6), 1263–1271. 10.1016/j.jhep.2011.02.033 PubMed Abstract | 10.1016/j.jhep.2011.02.033 | Google Scholar 21703205 PMC6541203

[B48] Reyes-Gordillo K. Shah R. Muriel P. (2017). Oxidative Stress and Inflammation in Hepatic Diseases: Current and Future Therapy. Oxid. Med. Cell Longev. 2017, 3140673. 10.1155/2017/3140673 PubMed Abstract | 10.1155/2017/3140673 | Google Scholar 28203318 PMC5292177

[B49] Rolando N. Wade J. Davalos M. Wendon J. Philpott-Howard J. Williams R. (2000). The Systemic Inflammatory Response Syndrome in Acute Liver Failure. Hepatology 32 (4 Pt 1), 734–739. 10.1053/jhep.2000.17687 PubMed Abstract | 10.1053/jhep.2000.17687 | Google Scholar 11003617

[B50] Rosenkranz K. Kumbruch S. Tenbusch M. Marcus K. Marschner K. Dermietzel R. (2012). Transplantation of Human Umbilical Cord Blood Cells Mediated Beneficial Effects on Apoptosis, Angiogenesis and Neuronal Survival after Hypoxic-Ischemic Brain Injury in Rats. Cell Tissue Res. 348 (3), 429–438. 10.1007/s00441-012-1401-0 PubMed Abstract | 10.1007/s00441-012-1401-0 | Google Scholar 22526623

[B51] Rubinstein P. Dobrila L. Rosenfield R. E. Adamson J. W. Migliaccio G. Migliaccio A. R. (1995). Processing and Cryopreservation of Placental/umbilical Cord Blood for Unrelated Bone Marrow Reconstitution. Proc. Natl. Acad. Sci. U. S. A. 92 (22), 10119–10122. 10.1073/pnas.92.22.10119 PubMed Abstract | 10.1073/pnas.92.22.10119 | Google Scholar 7479737 PMC40747

[B52] Ryu K. H. Kim S. Y. Kim Y. R. Woo S. Y. Sung S. H. Kim H. S. (2014). Tonsil-derived Mesenchymal Stem Cells Alleviate Concanavalin A-Induced Acute Liver Injury. Exp. Cell Res. 326 (1), 143–154. 10.1016/j.yexcr.2014.06.007 PubMed Abstract | 10.1016/j.yexcr.2014.06.007 | Google Scholar 24954408

[B53] Scaffidi P. Misteli T. Bianchi M. E. (2002). Release of Chromatin Protein HMGB1 by Necrotic Cells Triggers Inflammation. Nature 418 (6894), 191–195. 10.1038/nature00858 PubMed Abstract | 10.1038/nature00858 | Google Scholar 12110890

[B54] Schmidt-Arras D. Rose-John S. (2016). IL-6 Pathway in the Liver: From Physiopathology to Therapy. J. Hepatol. 64 (6), 1403–1415. 10.1016/j.jhep.2016.02.004 PubMed Abstract | 10.1016/j.jhep.2016.02.004 | Google Scholar 26867490

[B55] Shaw T. D. Krasnodembskaya A. D. Schroeder G. N. Zumla A. Maeurer M. O'Kane C. M. (2021). Mesenchymal Stromal Cells: an Antimicrobial and Host-Directed Therapy for Complex Infectious Diseases. Clin. Microbiol. Rev. 34 (4), e0006421. 10.1128/cmr.00064-21 PubMed Abstract | 10.1128/cmr.00064-21 | Google Scholar 34612662 PMC8510528

[B56] Starkey Lewis P. Campana L. Aleksieva N. Cartwright J. A. Mackinnon A. O'Duibhir E. (2020). Alternatively Activated Macrophages Promote Resolution of Necrosis Following Acute Liver Injury. J. Hepatol. 73 (2), 349–360. 10.1016/j.jhep.2020.02.031 PubMed Abstract | 10.1016/j.jhep.2020.02.031 | Google Scholar 32169610 PMC7378576

[B57] Tasso R. Pennesi G. (2009). When Stem Cells Meet Immunoregulation. Int. Immunopharmacol. 9 (5), 596–598. 10.1016/j.intimp.2009.01.014 PubMed Abstract | 10.1016/j.intimp.2009.01.014 | Google Scholar 19539568

[B58] Wang H. X. Liu M. Weng S. Y. Li J. J. Xie C. He H. L. (2012). Immune Mechanisms of Concanavalin A Model of Autoimmune Hepatitis. World J. Gastroenterol. 18 (2), 119–125. 10.3748/wjg.v18.i2.119 PubMed Abstract | 10.3748/wjg.v18.i2.119 | Google Scholar 22253517 PMC3257438

[B59] Weiss M. L. Troyer D. L. (2006). Stem Cells in the Umbilical Cord. Stem Cell Rev. 2 (2), 155–162. 10.1007/s12015-006-0022-y PubMed Abstract | 10.1007/s12015-006-0022-y | Google Scholar 17237554 PMC3753204

[B60] Wu Z. Han M. Chen T. Yan W. Ning Q. (2010). Acute Liver Failure: Mechanisms of Immune-Mediated Liver Injury. Liver Int. 30 (6), 782–794. 10.1111/j.1478-3231.2010.02262.x PubMed Abstract | 10.1111/j.1478-3231.2010.02262.x | Google Scholar 20492514

[B61] Xiang X. Gui H. King N. J. Cole L. Wang H. Xie Q. (2012). IL-22 and Non-ELR-CXC Chemokine Expression in Chronic Hepatitis B Virus-Infected Liver. Immunol. Cell Biol. 90 (6), 611–619. 10.1038/icb.2011.79 PubMed Abstract | 10.1038/icb.2011.79 | Google Scholar 21946664

[B62] Xiang X. Feng D. Hwang S. Ren T. Wang X. Trojnar E. (2020). Interleukin-22 Ameliorates Acute-On-Chronic Liver Failure by Reprogramming Impaired Regeneration Pathways in Mice. J. Hepatol. 72 (4), 736–745. 10.1016/j.jhep.2019.11.013 PubMed Abstract | 10.1016/j.jhep.2019.11.013 | Google Scholar 31786256 PMC7085428

[B63] Xu M. J. Feng D. Wang H. Guan Y. Yan X. Gao B. (2014). IL-22 Ameliorates Renal Ischemia-Reperfusion Injury by Targeting Proximal Tubule Epithelium. J. Am. Soc. Nephrol. 25 (5), 967–977. 10.1681/ASN.2013060611 PubMed Abstract | 10.1681/ASN.2013060611 | Google Scholar 24459233 PMC4005304

[B64] Yamanaka S. (2020). Pluripotent Stem Cell-Based Cell Therapy-Promise and Challenges. Cell Stem Cell 27 (4), 523–531. 10.1016/j.stem.2020.09.014 PubMed Abstract | 10.1016/j.stem.2020.09.014 | Google Scholar 33007237

[B65] Yang R. Tonnesseen T. I. (2019). DAMPs and Sterile Inflammation in Drug Hepatotoxicity. Hepatol. Int. 13 (1), 42–50. 10.1007/s12072-018-9911-9 PubMed Abstract | 10.1007/s12072-018-9911-9 | Google Scholar 30474802

[B66] Yi Y. Zhang W. Tao L. Shao Q. Xu Q. Chen Y. (2021). RIP1 Kinase Inactivation Protects against Acetaminophen-Induced Acute Liver Injury in Mice. Free Radic. Biol. Med. 174, 57–65. 10.1016/j.freeradbiomed.2021.07.034 PubMed Abstract | 10.1016/j.freeradbiomed.2021.07.034 | Google Scholar 34324981

[B67] You L. Wang Z. Li H. Shou J. Jing Z. Xie J. (2015). The Role of STAT3 in Autophagy. Autophagy 11 (5), 729–739. 10.1080/15548627.2015.1017192 PubMed Abstract | 10.1080/15548627.2015.1017192 | Google Scholar 25951043 PMC4509450

[B68] Yu H. Yuan X. Zhao M. Wang W. Gong D. (2021). Efficacy of Human Umbilical Cord Blood-Mononuclear Cell Transplantation for MSA Treatment and its Effects on Changes in T-Cell Subsets in Peripheral Blood and Inflammatory Factors. Dis. Markers 2021, 5290766. 10.1155/2021/5290766 PubMed Abstract | 10.1155/2021/5290766 | Google Scholar 34900026 PMC8654533

[B69] Zhang Y. Liu D. Hu H. Zhang P. Xie R. Cui W. (2019). HIF-1α/BNIP3 Signaling Pathway-Induced-Autophagy Plays Protective Role during Myocardial Ischemia-Reperfusion Injury. Biomed. Pharmacother. 120, 109464. 10.1016/j.biopha.2019.109464 PubMed Abstract | 10.1016/j.biopha.2019.109464 | Google Scholar 31590128

