# Supplementary material for: Human Umbilical Cord Blood Mononuclear Cells Ameliorate CCl4-Induced Acute Liver Injury in Mice via Inhibiting Inflammatory Responses and Upregulating Peripheral Interleukin-22
Source: Front Pharmacol. 2022 Jul 22;13:924464. doi: 10.3389/fphar.2022.924464 (PMC9356225; doi:10.3389/fphar.2022.924464)
Supplement: Supplementary file 1 [file DataSheet1.pdf]

Human umbilical cord blood mononuclear cells ameliorate CCl_4-induced acute liver injury in mice via inhibiting inflammatory responses and up-regulating interleukin-22

Jinming Zhang[1,2,†], Hengben Zhai[1,2,†], Pei Yu[3,†], Dabao Shang[1,2], Ruidong Mo[1,2], Ziqiang Li[1,2], Xiaolin Wang[1,2], Jie Lu[1,2,*], Qing Xie[1,2,*], Xiaogang Xiang[1,2,*]

Supplementary Materials:

Supporting Methods:

Identification of hUCBMNCs

The isolated hUCBMNCs were identified by the surface makers on cells in China National Institute for Food and Drug Control (NIFDC). The results of mRNA expression of surface markers were shown as follows (Table S1):

Table S1. Identification of surface markers on cells of hUCBMNCs.

Surface markers on cells	Percentage
$CD3^+$	34.8%
$CD3^+CD4^+$	28.2%
$CD3^-CD4^+$	13.2%
$CD3^+CD8^+$	5.8%
$CD3^-CD8^+$	1.3%
$CD4^+$	41.4%
$CD8^+$	7.2%
$CD14^+$	24.9%
$CD45RA^+CD4^+$	30.6%
$CD45RO^+CD4^+$	28.1%
$CD45RA^+CD8^+$	7.4%
$CD45RO^+CD8^+$	6.5%
$CD5^+$	41.2%
$CD19^+$	13.1%
$CD23^+$	undetermined
$CD1c^+$	13.8%
$CD56^+CD3^-$	1.8%
$CD56^+CD16^+$	1.0%
$CD56^+CD16^-$	0.9%
$CD38^+$	69.1%
$CD133^+$	2.3%
$CD34^+$	1.3%
$CD34^+CD133^+$	0.5%
$CD34^+CD133^-$	0.8%

Note: Surface markers of hUCBMNCs were identified by using PCR with major expression of CD38, CD4, CD5 and CD3 and no less than 1% of CD34.

Western blotting

Liver tissue was homogenized in lysis buffer to obtain total protein and protein concentration was determined by bicinchoninic acid (BCA) protein assay (Thermo Scientific™) [1]. Total protein subjected to SDS-PAGE (polyacrylamide gel electrophoresis) was analyzed qualitatively with specified antibodies, consisting of signal transducer and activator of transcription 3 (STAT3) antibody (Cell Signal Technology, 9139), phospho-STAT3 (p-STAT3) antibody (Cell Signal Technology, 9145), extracellular signal-regulated kinase (ERK1/2) antibody (Beyotime, AF1051), p-ERK1 antibody (Beyotime, AF1891), glyceraldehyde 3-phosphate dehydrogenase (GAPDH) antibody (Beyotime, AF1186), cleaved-caspase3 antibody (abcam, 21032), capase3 antibody (abcam, 32351), adenosine 5'-monophosphate-activated protein kinas (AMPKα) antibody (Cell Signal Technology, 2532), p-AMPKα antibody (Cell Signal Technology, 2535), LC3B (Sigama-aldrich, L7543), p62 antibody (Cell Signal Technology, 5114).

Real-time quantitative polymerase chain reaction (RT-qPCR)

Total messenger RNA (mRNA) was extracted from the frozen liver tissue according to the manufacturer's instructions (EZBioscience, Tisssue RNA Purification Kit, RN001). Reverse transcription of mRNA into cDNA was performed using Reverse Transcription Kit (with DNase) (EZBioscience, RT3). For each specific gene, primer pairs were as follows (Table S2):

Table S2. Mouse primers used in RT-qPCR analysis

Genes	Forward primers (5'-3')	Reverse primers (5'-3')
18s	GCAATTATTCCCCATGAACG	GGCCTCACTAAACCATCCAA
Il6	TAGTCCTTCCTACCCCAATTTCC	CTTGGTCCTTAGCCACTCCTTC
Tnfa	CAGGCGGTGCCTATGTCTC	CGATCACCCCGAAGTTCAGTAG
Il1b	TTCAGGCAGGCAGTATCACTC	GAAGGTCCACGGGAAAGACAC
Ifng	TCAGGTAGTAACAGGCTG TCC	CATTCGGGTGTAGTCACAGTT
Cxcl1	ACTGCACCCAAACCGAAGTC	TGGGGACACCTTTTAGCATCTT
Il10	CTTACTGACTGGCATGAGGATCA	GCAGCTCTAGGAGCATGTGG
Il17a	TCAGCGTGTCCAAACACTGAG	TCAGCGTGTCCAAACACTGAG

Ccr2	ATGCAAGTTCAGCTGCCTGC	ATGCCGTGGATGAACTGAGG
F4/80	GGAAAGCACCATGTTAGCTGC	CCTCTGGCTGCCAAGTTAATG
Cxcr1	ATGCCCTCTATTCTGCCAGAT	GTGCTCCGGTTGTATAAGATGAC
Il2	GTGCTCCTTGTCAACAGCG	GGGGAGTTTCAGGTTCCTGTA
Cxcl9	CCTAGTGATAAGGAATGCACGATG	CTAGGCAGGTTTGATCTCCGTTC
Cxcl10	ATCATCCCTGCGAGCCTATCCT	GACCTTTTTTGGCTAAACGCTTTC
Cxcl11	CCGAGTAACGGCTGCGACAAAG	CCTGCATTATGAGGCGAGCTTG
Duox2	TGACGGTGTTTATCAGGCTCT	TTTGCCCTTAGCGACAGCATC
Nox3	TGGCAGTAAACGCCTATCTGT	CGGAACCCAGAATAACTCGTGTA
Hif1a	TCTCGGCGAAGCAAAGAGTC	AGCCATCTAGGGCTTTCAGATAA
Binp3	CTGGGTAGAACTGCACTTCAG	GGAGCTACTTCGTCCAGATTCAT
Foxo1	CCCAGGCCGGAGTTTAACC	GTTGCTCATAAAGTCGGTGCT
Pik3c3	GGGCTATACCAAGAGACATGC	CGCCTTGTAGGATGTTCTGACT
Ctsb	CAGGCTGGACGCAACTTCTAC	TCACCGAACGCAACCCTTC
Becn1	ATGGAGGGGTCTAAGGCGTC	TGGGCTGTGGTAAGTAATGGA
Bnip3l	CTGGAGCACGTTCCTTCCTC	ACAGTGCGAACTGCCTCTTG

Hematoxylin & eosin (H&E) staining

Formalin-fixed mice liver tissues were processed, and paraffin-embedded sections of 4-µm thickness were stained with H&E for histological analysis [2]. Representative images were showed, and necrosis areas were quantified by Image J software (version 1.47v, NIH) in 5 random microscopic fields (×100) per sample.

BrdU staining

BrdU In-Situ Detection Kit II (BD Pharmingen™) was used for detection of proliferating and dividing cells by immunohistochemistry [3]. The number of BrdU-positive cells per 200× high-powered field were counted as previous [3].

Determination of serum interleukin 22

Mouse IL-22 (Interleukin 22) ELISA Kit (Elabscience, E-EL-M2446c) was used to detect the concentration of serum IL-22 according to the manufacturer's instruction.

Determination of malondialdehyde (MDA) in liver tissue

One of the end products of lipid peroxidation, malondialdehyde (MDA), was determined by using Lipid Peroxidation MDA Assay Kit (Beyotime, S0131) in liver homogenate supernatant (MDA/liver protein) [4].

Determination of multi-inflammatory cytokines in mouse serum

Mouse serum inflammatory cytokines were detected by flow cytometry using LEGENDplex^{TM} Mouse Inflammation Panel (Cat. No. 740446).

Supporting Figures

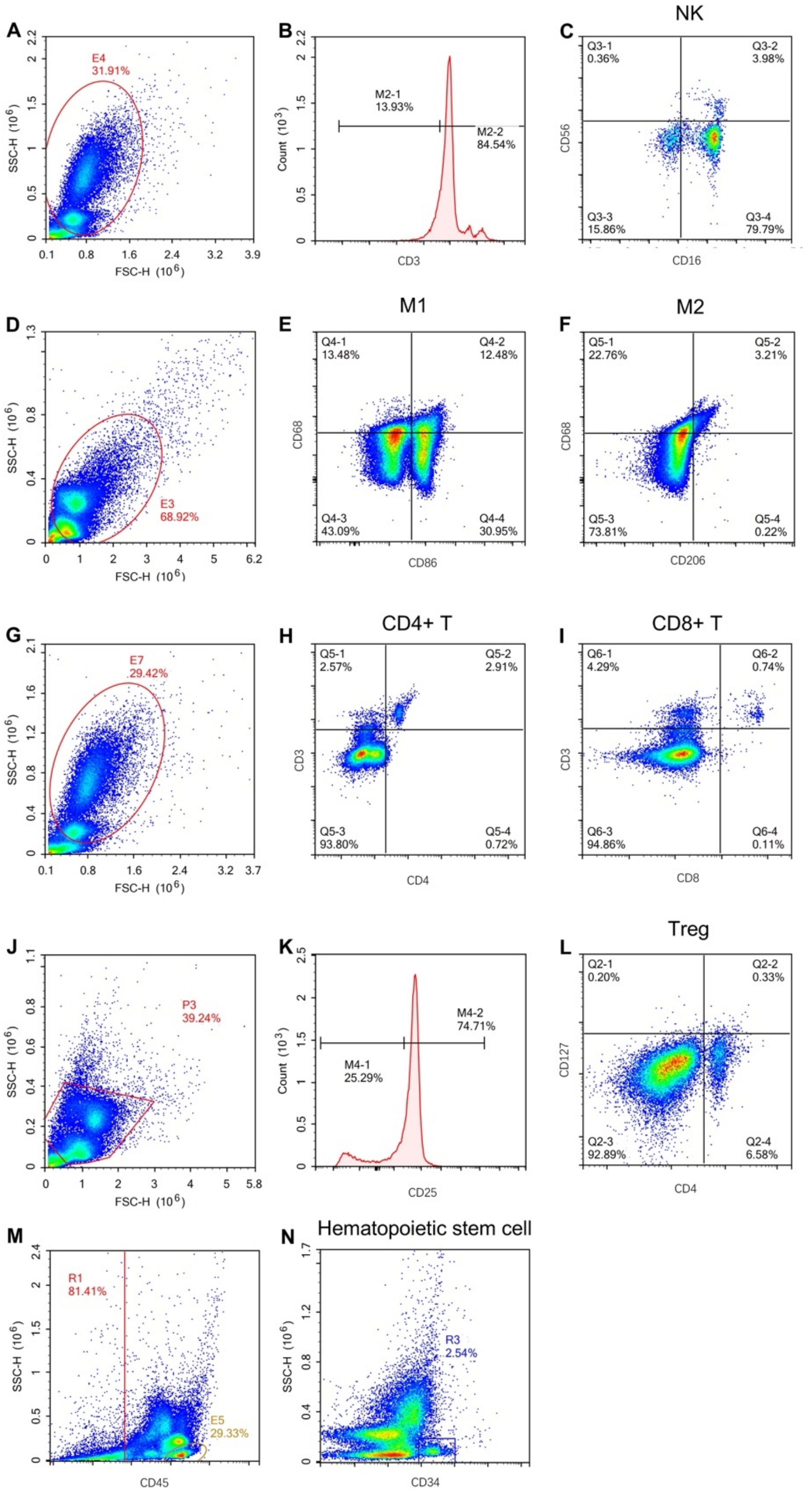

Figure S1. Typical cells subsets in hUCBMNCs.
(A, B, C) Representative flow plots of NK cells. **(D, E, F)** Representative flow plots of M1 and M2 macrophages. **(G, H, I)** Representative flow plots of $CD4^+$ T cells and $CD8^+$ T cells. (J, K, L) Representative flow plots of Treg cells. **(M, N)** Representative flow plots of hematopoietic stem cell.

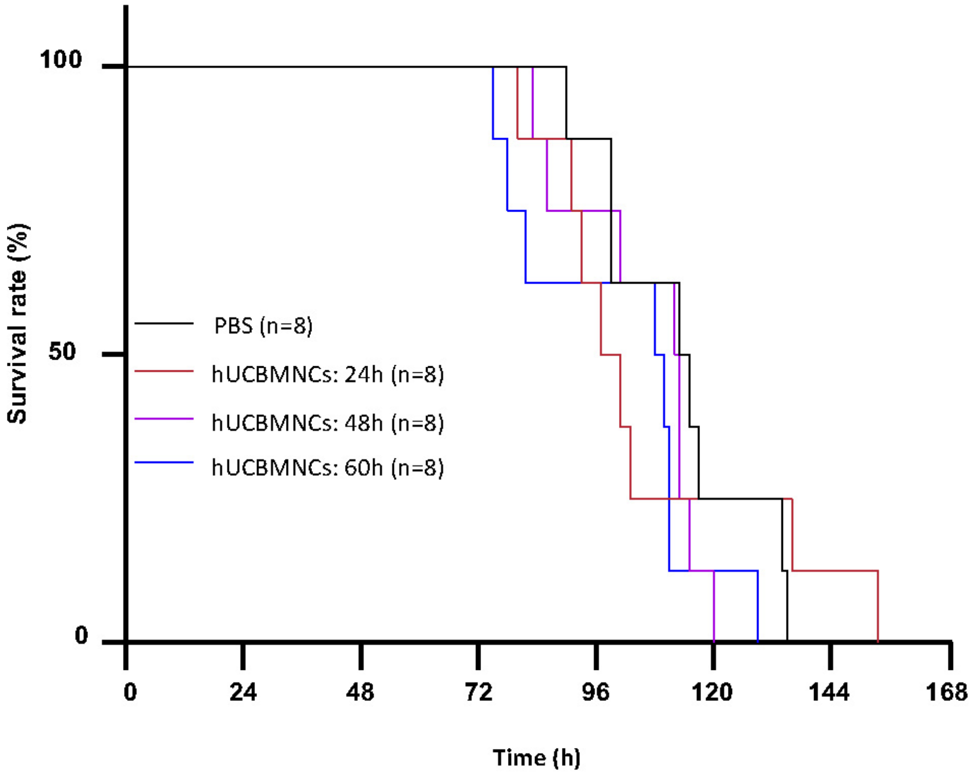

Figure S2. The survival rate of hUCBMNCs treatment in mice infected by *S. Typhimurium.*

S. Typhimurium (8×10^3 CFU/mouse) was intraperitoneally administrated at 0h and hUCBMNCs were applied at 24h, 48h, 60h (n=8/group).

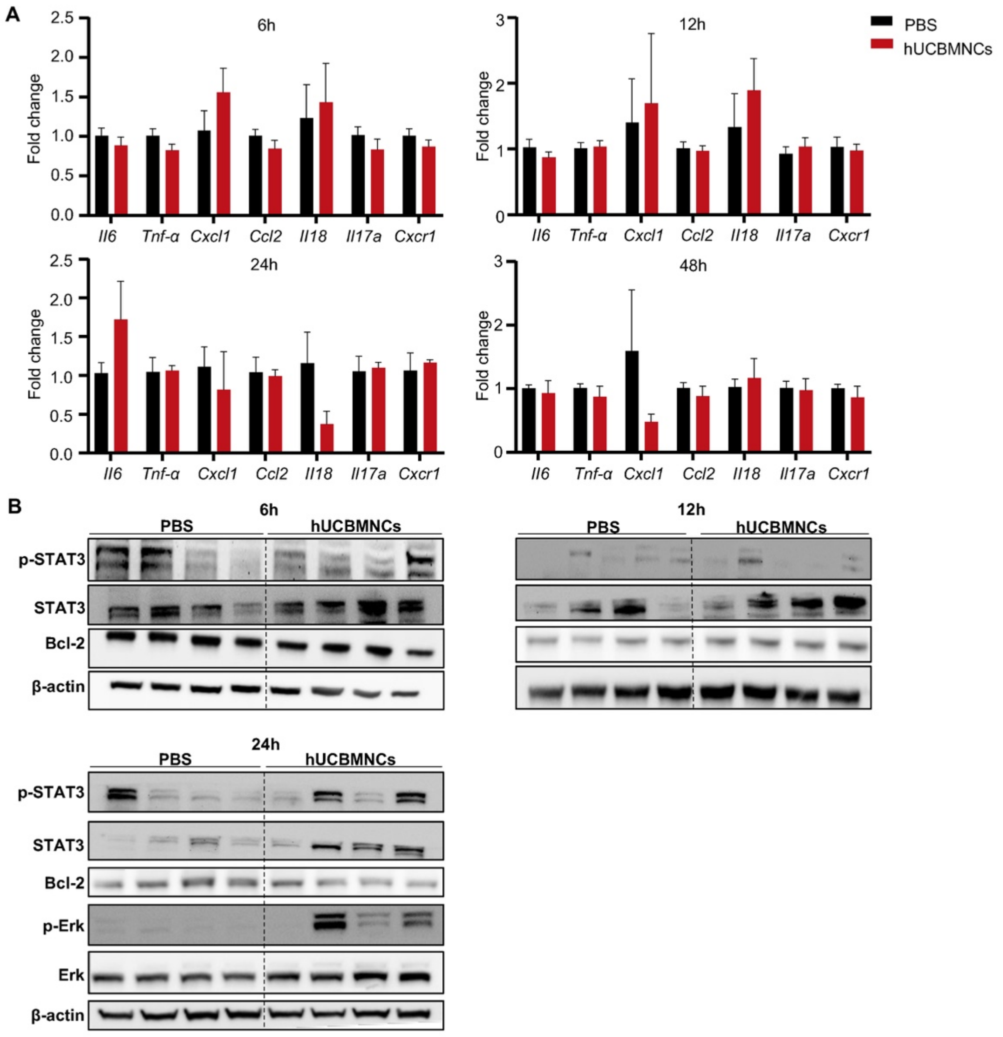

Figure S3. Relative mRNA expression levels of liver inflammatory markers and western blotting results of mice liver in APAP-induced acute liver injury.

(A) Relative mRNA expression levels of liver inflammatory markers (n=4/time point/group). **(B)** Western blotting of liver regeneration-related proteins (n=4/time point/group).

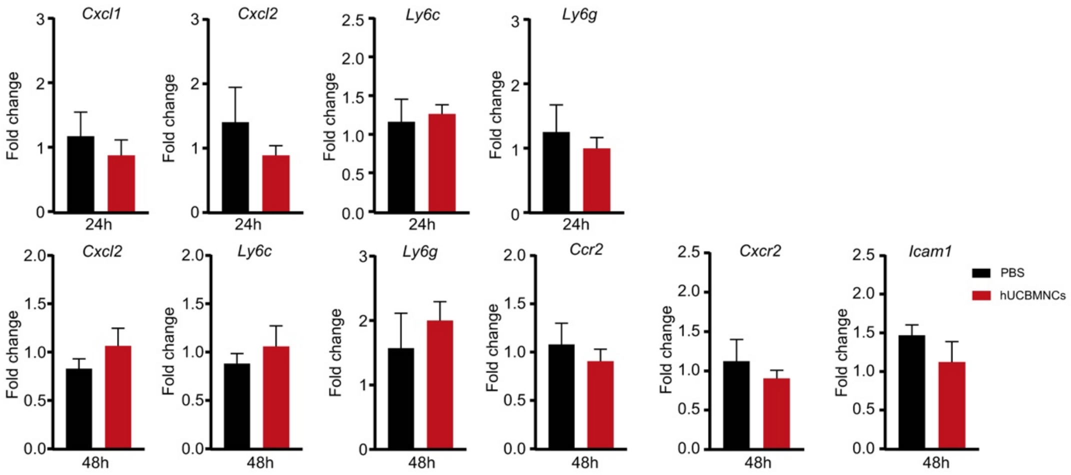

Figure S4. Hepatic mRNA levels of inflammatory markers of CCl_4-induced acute liver injury.

Hepatic mRNA levels of inflammatory markers in CCl_4-induced acute liver injury at 24h and 48h post CCl_4 insult (n=5/time point/group).

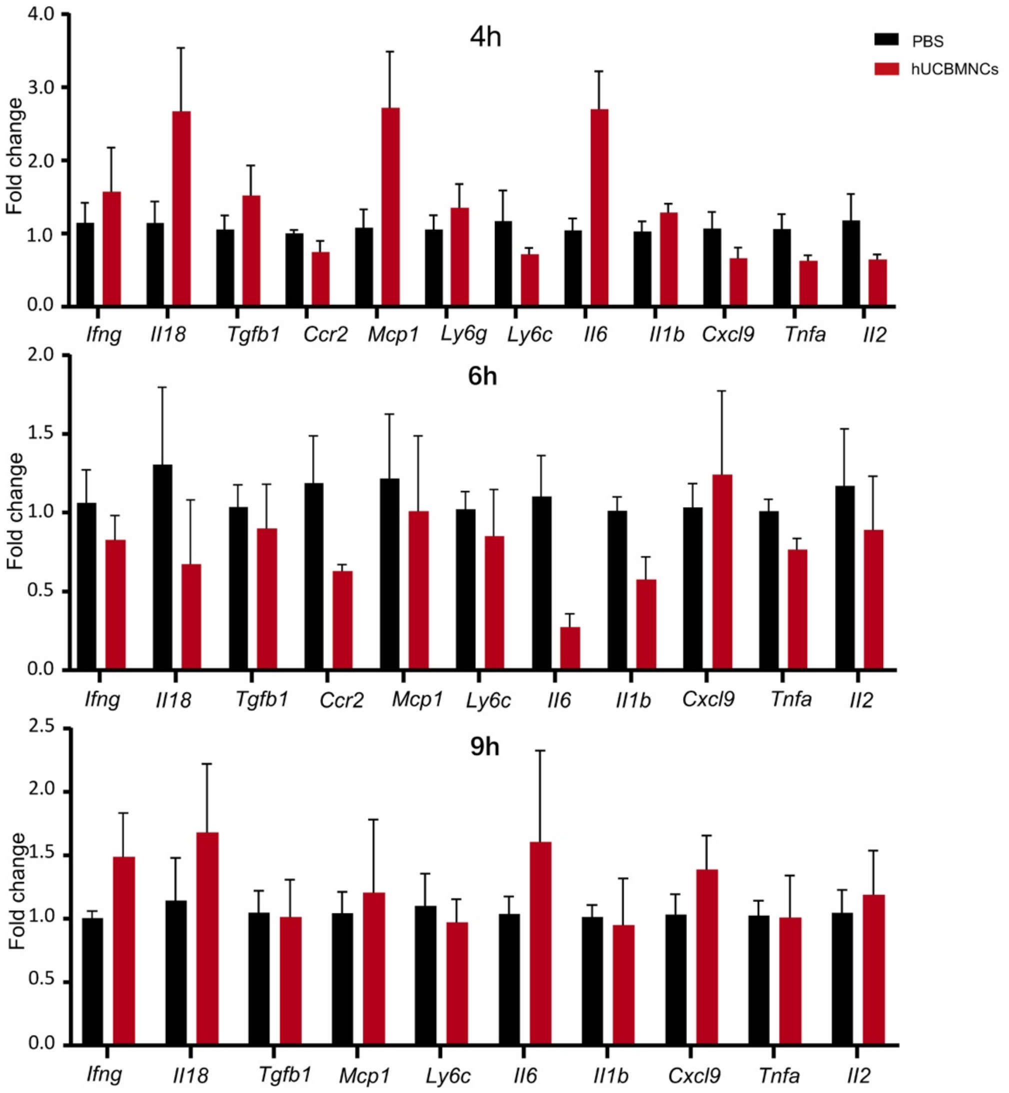

Figure S5. Relative mRNA expression levels of liver inflammatory markers.
Relative mRNA expression levels of liver inflammatory markers (n=4/time point/group) were shown at 4h, 6h and 9h post ConA insult.

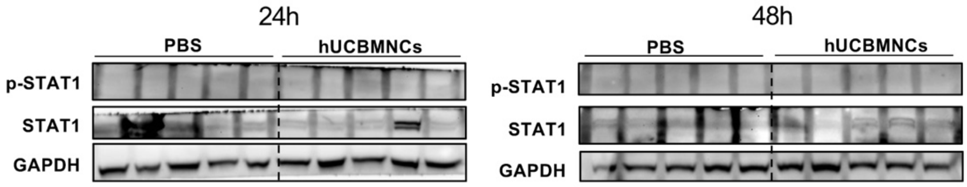

Figure S6. Western blotting results of mice liver in CCl_4-induced acute liver injury. Western blotting results of p-STAT1, STAT1 and GAPDH expression at 24h and 48h in mice liver.

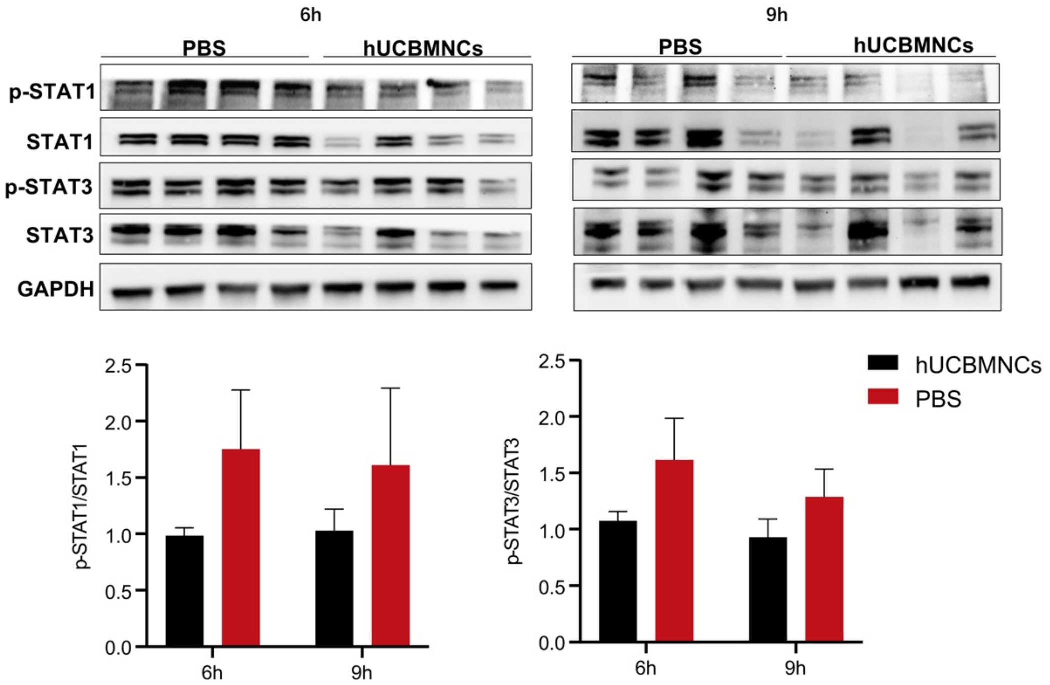

Figure S7. Western blotting results of mice live in ConA-induced acute liver injury. Western blotting results of p-STAT1, STAT1, P-STAT3, STAT3 and GAPDH expression in mice liver.

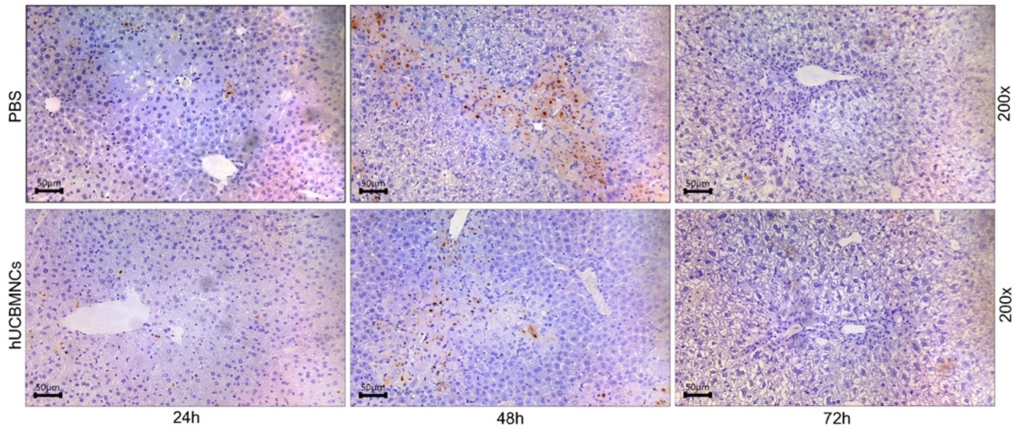

Figure S8. Hepatic mRNA levels of inflammatory markers and TUNEL staining of CCl_4-induced acute liver injury.

(TUNEL staining of liver tissue at 24h, 48h, 72h post CCl_4 insult (n=5/time point/group).

Supporting documents

Attachment 1.
Quality control results of hUCBMNCs by China National Institutes for Food and Drug Control (NIFDC) [The original language is Chinese and English translation is attached]

中国食品药品检定研究院检验报告

报告编号：SH202006758　　　　　　　　　　　　　　　　共3页，第1页

检品名称	脐带血单个核细胞	检品编号	SH0417202003155
供样单位	山东省齐鲁干细胞工程有限公司	菌号/代次	编号：RDCB0517
检品来源	山东省齐鲁干细胞工程有限公司	检品状态	液氮冻存
检验目的	合同检验	检品数量	10管
检验项目	部分检验	收样日期	2020年6月17日
检验依据	约定方法		

检验项目	标准规定	检验结果
【细胞鉴别试验】		
细胞形态检查	应为悬浮细胞，呈圆球形	符合规定
细胞株鉴别（人源STR图谱分析）	应为单一细胞来源	符合规定<br>AMEL:X;　vWA:14,18<br>D21S11:31,32.2;<br>D18S51:13,14<br>PentaE:16;　D5S818:11,13<br>D13S317:8;　D7S820:9,11<br>D16S539:11,12;　FGA:20,26<br>D3S1358:15,16;　TH01:8,9<br>D8S1179:14,15;　TPOX:8,10<br>CSF1PO:10,12;　PentaD:11,12
细胞种属鉴别（PCR法）	应为人源细胞	符合规定
细胞表面抗原分析		
$CD3^{+}$(%)	报告结果	34.8
$CD3^{+}CD4^{+}$(%)	报告结果	28.2
$CD3^{-}CD4^{+}$(%)	报告结果	13.2
$CD3^{+}CD8^{+}$(%)	报告结果	5.8
$CD3^{-}CD8^{+}$(%)	报告结果	1.3
$CD4^{+}$(%)	报告结果	41.4
$CD8^{+}$(%)	报告结果	7.2
$CD14^{+}$(%)	报告结果	24.9
$CD45RA^{+}CD4^{+}$(%)	报告结果	30.6
$CD45RO^{+}CD4^{+}$(%)	报告结果	28.1
$CD45RA^{+}CD8^{+}$(%)	报告结果	7.4
$CD45RO^{+}CD8^{+}$(%)	报告结果	6.5
$CD5^{+}$(%)	报告结果	41.2
$CD19^{+}$(%)	报告结果	13.1
$CD23^{+}$(%)	报告结果	未检出
$CD1c^{+}$(%)	报告结果	13.8

接下页

Inspection report of China National Institute for Food and Drug Control

Report Number: SH202006758

**Name of samples**	Human cord blood mononuclear cells	**Number of samples**	SH0417202003155
**Source of samples**	Shandong Cord Blood Bank	**Code**	RDCB0517
		**Condition of samples**	Frozen by liquid nitrogen
**Purpose of inspection**	Inspection according to the contract	**Amount of samples**	10 tubes
**Item of inspection**	Part of samples	**Time of collection**	17th June, 2020
**Standards of inspection**	Agreed method		

**Item of testing**	**Standard Order**	**Results of testing**
[Experiment of cell identification]		
Examination of cellular morphology	Suspended cells with spherical shape	Conformity
Identification of cell line (Analysis of human STR atlas)	Single cell source	Conformity<br>AMEL: X; vWA: 14,18<br>D21S11: 31, 32, 2<br>D18S51: 13, 14<br>PentaE: 16; D5S818: 11,13<br>D13S317: 8; D7S820: 9,11<br>D16S539: 11, 12; FGA: 20, 26<br>D3S1358: 15, 16; TH01: 8, 9<br>D8S1179: 14, 15; TP0X: 8, 9<br>CSFIP0: 10, 12; PentaD: 11, 12
Identification of cells (PCR)<br>Analysis of surface markers on cells		
$CD3^+$	Results of report	34.8%
$CD3^+CD4^+$	Results of report	28.2%
$CD3^-CD4^+$	Results of report	13.2%
$CD3^+CD8^+$	Results of report	5.8%
$CD3^-CD8^+$	Results of report	1.3%
$CD4^+$	Results of report	41.4%
$CD8^+$	Results of report	7.2%
$CD14^+$	Results of report	24.9%
$CD45RA^+CD4^+$	Results of report	30.6%
$CD45RO^+CD4^+$	Results of report	28.1%
$CD45RA^+CD8^+$	Results of report	7.4%
$CD45RO^+CD8^+$	Results of report	6.5%
$CD5^+$	Results of report	41.2%
$CD19^+$	Results of report	13.1%
$CD23^+$	Results of report	undetermined
$CD1c^+$	Results of report	13.8%

Continued

中国食品药品检定研究院检验报告

接上页

检验项目	标准规定	检验结果
$CD56^+CD3^-$(%)	报告结果	1.8
$CD56^+CD16^+$(%)	报告结果	1.0
$CD56^+CD16^-$(%)	报告结果	0.9
$CD38^+$(%)	报告结果	69.1
$CD133^+$(%)	报告结果	2.3
$CD34^+$(%)	报告结果	1.3
$CD34^+CD133^+$(%)	报告结果	0.5
$CD34^+CD133^-$(%)	报告结果	0.8
【无菌检查】		
薄膜过滤法	应无菌生长	符合规定
【支原体检查】		
指示细胞培养法	应为阴性	符合规定
【内毒素检测】		
动态显色法 (EU/ml)	<0.25	符合规定
【特殊人源病毒检查】		
人免疫缺陷病毒Ⅰ型(HIV-1)	应为阴性	符合规定
人乙型肝炎病毒(HBV)	应为阴性	符合规定
人丙型肝炎病毒(HCV)	应为阴性	符合规定
人巨细胞病毒(HCMV)	应为阴性	符合规定
人EB病毒(EBV)	应为阴性	符合规定
人细小病毒B19	应为阴性	符合规定
人疱疹病毒6型、7型(HHV-6，HHV-7)	应为阴性	符合规定
人乳头瘤病毒(HPV)	应为阴性	符合规定
【免疫学反应检测】		
淋巴细胞增殖抑制试验※※		
淋巴细胞增殖抑制率	报告结果	待检细胞与外周血单个核细胞(PBMC)以1:1比例共培养，淋巴细胞增殖比例由97.32%降低为96.57%；待检细胞对淋巴细胞增殖的抑制率为0.8%
特定淋巴细胞亚群检测※※		
Th1增殖抑制率	报告结果	待检细胞与PBMC以1:1比例共培养，对Th1增殖无抑制作用
Th17增殖抑制率	报告结果	待检细胞与PBMC以1:1比例共培养，对Th17增殖无抑制作用

接下页

Continued

**Items of testing**	**Standard Order**	**Results of testing**
$CD56^+CD3^-$	Results of report	1.8%
$CD56^+CD16^+$	Results of report	1.0%
$CD56^+CD16^-$	Results of report	0.9%
$CD38^+$	Results of report	69.1%
$CD133^+$	Results of report	2.3%
$CD34^+$	Results of report	1.3%
$CD34^+CD133^+$	Results of report	0.5%
$CD34^+CD133^-$	Results of report	0.8%
**[Sterility test]**<br>Membrane filtration methods	Sterile	Conformity
**[Testing for mycoplasma]**<br>Indicated cell culture method	Negative	Conformity
**[Testing for endotoxin]**<br>Dynamic color rendering (EU/ml)	< 0.25	Conformity
**[Testing for special pathogen from human]**		
Human immunodeficiency virus type 1 (HIV-1)	Negative	Conformity
Human hepatitis B virus (HBV)	Negative	Conformity
Human hepatitis C virus (HCV)	Negative	Conformity
Human cytomegalovirus (HCMV)	Negative	Conformity
Human Epstein-Barr virus (EVB)	Negative	Conformity
Human parvovirus B19	Negative	Conformity
Human herpesvirus type 6 and type 7 (HHV-6, HHV-7)	Negative	Conformity
Human papilloma virus (HPV)	Negative	Conformity
**[Determination of immunological reaction]**<br>Inhibition test of lymphocyte proliferation**<br>Inhibition rate of lymphocyte proliferation	Results of report	The cells to be determined were co-cultured with peripheral blood mononuclear cells (PBMC) with quantity ratio 1:1. The proliferation rate of lymphocyte decreased from 97.32% to 96.57%. The inhibition rate of lymphocyte proliferation was 0.8%.
Detection of specific lymphocyte subsets**<br>Inhibition rate of Th1	Results of report	The cells to be determined were co-cultured with PBMC with quantity ratio 1:1. There was no effects of inhibition on Th1.
Inhibition rate of Th17	Results of report	The cells to be determined were co-cultured with PBMC with quantity ratio 1:1. There was no effects of inhibition on Th17.

Continued

中国食品药品检定研究院检验报告

报告编号：SH202006758

接上页

检验项目	标准规定	检验结果
Treg增殖促进率	报告结果	待检细胞与PBMC以1:1比例共培养，Treg细胞比例由2.80%升高为3.85%；待检细胞对Treg增殖的促进率为37.5%
淋巴细胞分泌TNF-α抑制试验※※	报告结果	待检细胞与PBMC以1:1比例共培养，对TNF-α分泌的抑制率为57.1%
【诱导分化能力检测】		
集落形成试验※※		
CFU-E集落数（个/10万个待检细胞）	报告结果	35
BFU-E集落数（个/10万个待检细胞）	报告结果	55
CFU-GEMM集落数（个/10万个待检细胞）	报告结果	15
CFU-GM集落数（个/10万个待检细胞）	报告结果	55
【细胞存活率检测】		
细胞存活率（%）	报告结果	68.5
【成瘤性检查】		
软琼脂克隆形成试验	应无克隆形成	符合规定

以下空白

备注：检验项目名称右上角带“※※”的为不在我院CNAS认可范围内。合同检验是在双方自愿基础上，按照合同约定开展的样品检验。

检验结论	本品按约定方法检验上述项目，结果如上所述。		
授权签字人	徐苗	签发日期	2020年11月27日

		**Continued**
**Items of testing**	**Standard Order**	**Results of testing**
Proliferation promotion rate of Treg	Results of report	The cells to be determined were co-cultured with PBMC with quantity ratio 1:1. The proportion of Treg cells increased from 2.8% to 3.85%. The promoting rate of Treg proliferation was 37.5%.
TNF-α inhibition test**	Results of report	The cells to be determined were co-cultured with PBMC with quantity ratio 1:1. The inhibition rate of TNF-α secretion was 57.1%.
**[Testing for potential of differentiation by inducing]** Testing for Colony formation**		
The number of CFU-E colony (CFU/10^5cells)	Results of report	35
The number of BFU-E colony (CFU/10^5cells)	Results of report	55
The number of CFU-GEMM colony (CFU/10^5cells)	Results of report	15
The number of CFU-GM colony (CFU/10^5cells)	Results of report	55
**[Testing for cell viability]** Cell viability (%)	Results of report	68.5
**[Examination of tumorigenesis]** Soft agar colony formation assay	No cell clone observed	Conformity
		**End**

Attachment 2.

Ethics Approval from the Institutional Animal Care and Use Committee of Ruijin Hospital, Shanghai Jiao Tong University School of Medicine

Ethics Approval from the Institutional Animal Care and Use Committee of Ruijin Hospital, Shanghai Jiao Tong University School of Medicine

**Experiment Title:**<br>The role and mechanism of hUCBMNCs in Hepatotoxin-mediated Hepatitis and T/NKT Cell Hepatitis in Mice
**Applicant:** Xiaogang Xiang
**Approval Comments**
We have the subject of "The role and mechanism of hUCBMNCs in Hepatotoxin-mediated Hepatitis and T/NKT Cell Hepatitis in Mice". The subject is approved by the Institutional Animal Care and Use Committee of Ruijin Hospital. The content and project design of the research basically conform to the ethical standards. We agree with examination and approval. We will conduct a review of animal ethics in strict accordance with the regulation related to the management of medical ethics and conduct research under the supervision of the Institutional Animal Care and Use Committee of our unit to ensure that the research accords with the relevant provisions of the Institutional Animal Care and Use Committee and safeguard the rights and interests of the animals.<br><br>The Institutional Animal Care and Use Committee of Ruijin Hospital Shanghai Jiao Tong University School of Medicine<br>March 2, 2020<br>实验医学研究中心

Reference

1. Schretter, C.E., et al., *A gut microbial factor modulates locomotor behaviour in Drosophila.* Nature, 2018. **563**(7731): p. 402-406.
2. Xu, M.J., et al., *Liver is the major source of elevated serum lipocalin-2 levels after bacterial infection or partial hepatectomy: a critical role for IL-6/STAT3.* Hepatology, 2015. **61**(2): p. 692-702.
3. Hu, B.L., et al., *Interleukin-22 ameliorates liver fibrosis through miR-200a/beta-catenin.* Sci Rep, 2016. **6**: p. 36436.
4. Xu, S., et al., *Cadmium induced Drp1-dependent mitochondrial fragmentation by disturbing calcium homeostasis in its hepatotoxicity.* Cell Death Dis, 2013. **4**(3): p. e540.
